# Effects of drying time on physicochemical properties and volatile organic compounds of pork jerky revealed by GC-IMS and interpretable machine learning

**DOI:** 10.1016/j.fochx.2026.104562

**Published:** 2026-10-08

**Authors:** Xinyu Chen, Haoran Huang, Huangyu Chen, Siying Wang, Zeyuan Zang, Yujia Huo, Zhixin Xiong, DaoLi Lu, Yan Li

**Affiliations:** a School of Photoelectric Engineering of Changzhou Institute of Technology, Changzhou, Jiangsu 213002, China; b College of Chemical Engineering, Xiangtan University, Xiangtan 411105, China; c Shandong Hanon Science Instrument Co., Ltd, Dezhou, Shandong 253000, China; d College of Light Industry and Food Engineering, Nanjing Forestry University. Nanjing, Jiangsu 210037, China; e State Key Laboratory for Development and Utilization of Forest Food Resources, Nanjing Forestry University, Nanjing 210037, China; f School of Food and Biological Engineering, Jiangsu University, Zhenjiang 212013, China

**Keywords:** Pork jerky, GC-IMS, Volatile organic compounds (VOCs), Support vector regression (SVR), Extra trees regression (ETR), SHapley Additive exPlanations (SHAP)

## Abstract

Drying time affects dehydration, color development, Maillard reactions, and volatile organic compounds (VOCs) in pork jerky. Pork jerky was dried at 80 °C for 4–6 h, with marinated undried samples as the RAW control. Physicochemical properties, free amino acids, and volatile fingerprints were evaluated. Gas chromatography-ion mobility spectrometry (GC-IMS) detected 53 volatile signals, whose profiles changed markedly during drying. Support vector regression (SVR) and extra trees regression (ETR) were developed to predict moisture content, absorbance at 420 nm, fluorescence intensity, and L*, a*, and b* values from GC-IMS. Both models achieved strong test-set performance, with R^2^ values of 0.996–0.998 for SVR and 0.989–0.995 for ETR. SHapley Additive exPlanations (SHAP) analysis identified shared and model-specific volatile markers, including pentanal, furaneol, ethyl propanoate, heptanal, 2-hexenal, 2-butanone, and 3-isobutyl-2-methoxypyrazine. These results demonstrate the potential of GC-IMS combined with interpretable machine learning for quality prediction and volatile-marker screening during pork jerky drying.

## 1 Introduction

Pork jerky is a traditional dried meat product valued for its characteristic roasted-meaty flavor, firm texture, portability, and extended shelf life (Wu et al., 2025; Zhang et al., 2026). Its manufacture generally involves slicing, marination, hot-air drying, roasting, and packaging, among which drying is a decisive stage for establishing product stability and sensory quality (Keong et al., 2024; Pushparaj et al., 2025). Drying time determines not only the extent of moisture removal but also the duration for which proteins, lipids, reducing sugars, amino acids, and seasoning-derived components are exposed to heat (Shi, Feng, et al., 2021; Wang et al., 2020). Insufficient drying may leave excessive residual moisture and limit the development of the characteristic texture and flavor of pork jerky, whereas prolonged drying can increase shear force, intensify color deterioration, and produce an excessively dry and tough product (Kim et al., 2021; Shi, Zhao, et al., 2021). Therefore, controlling drying time is essential for balancing dehydration, flavor formation, and overall product quality.

The physicochemical and volatile changes occurring during drying are closely interconnected. As water is progressively removed, the food matrix contracts, water activity changes, and nonvolatile flavor precursors become concentrated (Lewicki, 2006). These changes alter mass transfer and the reaction environment, thereby affecting lipid oxidation, Maillard reactions, Strecker degradation, and the formation or loss of volatile compounds (Martins et al., 2000). The resulting volatile organic compounds (VOCs) formed during drying, including aldehydes, alcohols, ketones, acids, esters, furans, and pyrazines, may undergo concurrent formation, transformation, release, and loss (Wang et al., 2021). Consequently, the observed GC-IMS signals reflect the net balance among these processes. Many aldehydes, alcohols, and ketones originate from lipid oxidation, whereas pyrazines and selected furans and carbonyl compounds are mainly associated with sugar degradation, Maillard reactions, and Strecker degradation (Wang, Wang, et al., 2023). However, the origin of furans is compound-dependent, as some species, such as 2-pentylfuran, can also be generated through lipid oxidation. Free amino acids contribute directly to taste and also serve as precursors of Strecker aldehydes and nitrogen-containing heterocyclic compounds (Hofmann & Schieberle, 2000). Accordingly, changes in VOC fingerprints should be interpreted alongside moisture loss, browning-related indices, fluorescent Maillard intermediates, and CIE L*, a*, and b* color parameters.

Drying time can exert complex effects on VOC profiles because the formation, transformation, and loss of individual compounds do not necessarily proceed at comparable rates (Wang, Wang, et al., 2023). The measured abundance of a VOC reflects the net balance among precursor conversion, secondary reactions, volatilization, and thermal degradation; consequently, individual compounds may accumulate initially and subsequently decline, whereas others may continue to increase during prolonged heating (Wang et al., 2020). Conventional measurements of moisture, color, and Maillard related indices characterize important aspects of product quality but cannot fully resolve the multidimensional chemical changes underlying VOC evolution. Comprehensive volatile fingerprinting combined with multivariate modeling is therefore required to characterize temporal VOC trajectories and evaluate their quantitative associations with physicochemical quality.

Gas chromatography-ion mobility spectrometry (GC-IMS) has been increasingly applied to food-flavor analysis because it combines chromatographic separation with rapid ion-mobility detection (Huang et al., 2025). The technique offers high sensitivity, short analysis time, limited sample preparation, and intuitive fingerprint visualization (Huang et al., 2024). These characteristics are advantageous for comparing complex VOCs profiles among meat products subjected to different processing conditions. GC-IMS has been used to investigate volatile changes in beef, chicken, and other thermally processed foods (Chen et al., 2025; Jiang et al., 2025; Pan et al., 2025). Although GC-IMS has been increasingly applied to food and meat products, a substantial proportion of previous studies have emphasized VOCs identification, fingerprint visualization, univariate comparison, and PCA-based discrimination among sample groups (Wang et al., 2020; Wu et al., 2026). These approaches are effective for characterizing overall differences in volatile profiles but provide limited quantitative information on the relationships between correlated VOC signals and multiple continuous physicochemical indices (Yu et al., 2025). Interpretable regression studies simultaneously linking GC-IMS VOC fingerprints with moisture content, Maillard related indicators, and color parameters during pork jerky drying remain scarce. Therefore, further integration of GC-IMS with multivariate regression and model-interpretation methods is required to extract quality-related predictive information from complex VOC fingerprints.

Machine-learning regression provides a means of extracting latent predictive information from multivariate GC-IMS fingerprints. VOC variables may exhibit substantial correlation because their abundances are governed by shared precursors and interconnected chemical pathways, while their associations with physicochemical indices can be nonlinear (Chen et al., 2021; Li et al., 2025). Support vector regression (SVR) was selected because its regularized learning framework and kernel mapping enable nonlinear relationships to be modeled while controlling model complexity (Huang et al., 2022). Extra trees regression (ETR), in contrast, constructs an ensemble of strongly randomized regression trees by introducing randomness into both feature and split-point selection, thereby capturing nonlinear responses, threshold effects, and interactions among VOC variables (Wang, Mu, et al., 2023). SVR and ETR therefore represent distinct but potentially complementary modeling strategies: SVR estimates a regularized global relationship in a kernel-defined feature space, whereas ETR describes the response through randomized local partitions of the predictor space. Comparing the two algorithms allowed the predictive information contained in the GC-IMS VOC fingerprints to be evaluated across different nonlinear model structures.

High predictive accuracy alone does not reveal which VOC variables contribute most strongly to model outputs. Moreover, model-specific importance measures often provide only global rankings and may not indicate the direction or magnitude of feature contributions for individual samples. SHapley Additive exPlanations (SHAP) provides a unified additive feature-attribution framework that decomposes each prediction into a baseline value and the contributions of individual input variables (Lin et al., 2025). Mean absolute SHAP values can be used to quantify global feature importance, whereas the sign and distribution of sample-level SHAP values, interpreted together with the corresponding feature values, indicate whether a VOC signal shifts the predicted physicochemical index above or below the model baseline (Yan et al., 2024). Applying the same SHAP framework to both SVR and ETR facilitates the comparison of shared important VOCs and model-specific features across distinct nonlinear modeling structures. Importantly, SHAP explains the behavior of the fitted models and does not establish causal relationships between VOCs and physicochemical changes.

Accordingly, this study investigated the effects of drying time on the physicochemical properties, free amino acid profiles, and GC-IMS VOC fingerprints of pork jerky under a fixed drying temperature of 80 °C, which was selected as the standardized processing condition according to the industrial production procedure. Marinated undried samples and samples dried for 4, 4.5, 5, 5.5, and 6 h were systematically analyzed for moisture content, absorbance at 420 nm, fluorescence intensity, L*, a*, and b* values, free amino acids, and VOCs profiles. SVR and ETR models were subsequently developed to predict each physicochemical index independently from the GC-IMS VOC fingerprints, and SHAP analysis was subsequently used to quantify the contributions of individual VOC features to the model predictions and to identify the most influential quality-associated VOC variables. This integrated strategy aimed to elucidate the associations between VOCs evolution and physicochemical changes during drying and to assess the consistency of quality-associated VOCs identified by two complementary nonlinear regression models.

## 2 Materials and methods

### 2.1 Materials and reagents

Fresh pork hind legs were purchased from local supermarkets, stored at 4 °C, and promptly transported to the laboratory. Fish sauce, monosodium glutamate (provided by Lotus Health Group), sucrose (purchased from COFCO Group Co., Ltd.), pepper powder, eggs, and other ingredients were obtained from local supermarkets. All food-grade raw materials complied with national food hygiene regulations of China.

Trichloroacetic acid (analytical grade) was purchased from Sinopharm Chemical Reagent Co., Ltd. The calibration standards used for GC-IMS qualitative analysis included 2-nonanone (CAS: 821-55-6), 2-octanone (CAS: 111-13-7), 2-heptanone (CAS: 110-43-0), 2-hexanone (CAS: 591-78-6), 2-pentanone (CAS: 107-87-9), and 2-butanone (CAS: 78-93-3), all of which were purchased from China National Chemical Reagent Shanghai Co., Ltd.

### 2.2 Instrument and equipment

Electric heating constant temperature drying oven (Shanghai Jinghong Experimental Equipment Co., Ltd.), oven (Toshiba D132A1), colorimeter (Konica Minolta CR-400), wireless thermometer (Model Bai'ao 9929), card fluorophotometer (Varian Co., Ltd.), T6 ultraviolet spectrophotometer (Beijing Puxie General Instrument Co., Ltd.), and automatic amino acid analyzer (Sykam S-433D). Gas phase ion mobility spectrometry instrument (FlavourSpec© 1H1-00128)， which was equipped with an automatic sampler unit (PAL, Analytics AG, Zwingen, Switzerland) for 60 vials and furnished with an l mL Hamilton syringe. The device was also provided with a radioactive ionization source (tritium). The gas chromatographic column of the instrument is non-polar column SE-54-CB-1 quartz capillary column (constituted by 94% methyl-5% phenyl-1% vinyl silicone with 15 m of length, an internal diameter of 0.32 mm and 0.25 μm of film thickness).

### 2.3 Preparation of pork jerky samples

Fresh pork hind legs were purchased from a local supermarket on three consecutive days, with one hind leg obtained on each day. Each hind leg weighed approximately 8 kg before deboning and yielded approximately 5.1 kg of meat. Because the animals purchased on different days could not be confirmed to originate from the same slaughter batch, each procurement day, corresponding to one hind leg, was regarded as an independent biological replicate. Thus, three biological replicates were included at the raw-material level. The hind legs were transported to the laboratory under refrigerated conditions and processed separately to prevent cross-mixing among biological replicates.

After deboning, the meat was sliced parallel to the muscle-fiber direction into pieces approximately 2 mm thick. The formulation and processing procedure were provided by Shuangyu Food Co., Ltd. For each 1000 g of sliced pork, 125 g of sucrose, 4.7 g of monosodium glutamate, 0.3 g of pepper powder, 82.08 g of fish sauce, and 25.9 g of liquid egg white were added. The meat and seasonings were thoroughly mixed and marinated at 4 °C for 0.5 h. Preliminary drying trials were first conducted over a broader temperature range to characterize the general moisture-loss behavior of pork jerky. Based on the production conditions provided by Shuangyu Food Co., Ltd., 80 °C was subsequently fixed as the standardized drying temperature for all formal physicochemical, free amino acid, VOCs, sensory, and modeling experiments. The purpose of the formal experiment was therefore to investigate the effect of drying time at 80 °C rather than to optimize drying temperature. The marinated slices were spread evenly in a single layer on round bamboo trays without overlapping and were then transferred to a hot-air drying oven. The air temperature was monitored using a wireless temperature probe positioned approximately 5 mm above the sample surface. Drying was initiated after the measured temperature remained within ±1 °C of the set temperature for at least 3 min. Five drying-time treatments were established at 4, 4.5, 5, 5.5, and 6 h. The formal sampling scheme was intentionally concentrated within this later drying window to characterize quality evolution as the product approached its final low-moisture state, rather than to establish a complete kinetic profile covering the entire drying process. A separate tray of pork slices was used for each treatment to avoid disturbance caused by repeated sampling during drying. Samples collected immediately after marination and before hot-air drying were designated as the RAW control. After drying, the samples were cooled to room temperature and stored at 4 °C until physicochemical, free amino acid, and GC-IMS analyses.

For each treatment group, including RAW, 4, 4.5, 5, 5.5, and 6 h, 30 sample units were obtained according to a hierarchical design comprising three biological replicates, two independent processing batches within each biological replicate, and five sampling units from each batch. Consequently, 180 sample units were included in the endpoint dataset. The 30 sample units in each treatment group were not regarded as independent biological replicates but as sampling units nested within three biological replicates and their respective processing batches. For GC-IMS analysis, each sample unit was measured three times, and the averaged spectrum was used for subsequent data analysis.

### 2.4 Moisture content detection

Moisture content was determined according to the direct drying method specified in GB 5009.3–2016, Determination of Moisture in Foods. Approximately 10 g of each pork jerky sample was ground and placed in a pre-dried aluminum weighing container of constant mass. The sample was dried in a hot-air oven at 103 °C until the difference between two consecutive weighings was less than 2 mg. Moisture content was calculated from the mass loss during drying. Each measurement was performed in triplicate.

### 2.5 Color detection

Color measurements were performed using a calibrated colorimeter (Konica Minolta CR-400, Japan) under illuminant D65, with a 10° standard observer and an 8 mm aperture. Each sample was allowed to bloom for 20 min at 25 °C before measurement. The L*, a*, and b* values of the sample surface were then measured according to the CIE L*a*b* color system. To minimize measurement error, each sample was tested at least six times (Geng et al., 2015).

### 2.6 Maillard reaction detection

The method used to evaluate Maillard reaction-related changes was adapted with minor modifications from Geng (Geng et al., 2019). A 2.0 g portion of ground sample was accurately weighed and homogenized with 10 mL of deionized water. Then, 10 mL of cold 20% trichloroacetic acid was mixed with the homogenate. The mixture was centrifuged at 4 °C and 6000 r/min for 10 min, and the supernatant was subsequently filtered through filter paper for further analysis.

Following Matiacevich (Matiacevich & Buera, 2006), luorescence intensity was measured at an excitation wavelength of 370 nm and an emission wavelength of 440 nm as an indicator of Maillard reaction progression. Browning intensity was evaluated by measuring the absorbance of the filtrate at 420 nm according to Miśkiewicz (Miśkiewicz et al., 2018).

### 2.7 Free amino acid detection

Free amino acids were determined using a method adapted with minor modifications from Geng (Geng et al., 2018). Briefly, 3.0 g of pork jerky sample from each drying treatment was homogenized with 10 mL of deionized water. Then 10 mL of cold 10% trichloroacetic acid solution was added to the homogenate, and the mixture was centrifuged at 4 °C with 8000 r/min for 10 min. After centrifugation, the supernatant was collected, and the precipitate was washed three times with 5% trichloroacetic acid. The combined filtrate was passed through a 0.45 μm membrane before analysis. Free amino acid contents were determined using an amino acid analyzer equipped with a PEEK separation column (4.6 × 150 mm, 7 μm).

To eliminate the direct effect of differences in moisture content among drying treatments, the FAA contents were converted to a dry-matter basis using the moisture content measured for the corresponding samples. The dry-basis FAA content was calculated as follows:

(1) $$C_{\mathrm{dry}}=C_{\text{sample}}/\left(1-\frac{M}{100}\right)$$

where $C_{\mathrm{dry}}$ is the FAA content expressed as mg/100 g dry weight, $C_{\text{sample}}$ is the FAA content calculated on the original sample-mass basis, and M is the measured moisture content (%) of the corresponding sample. All FAA results are therefore reported as mg/100 g dry weight.

### 2.8 Volatile organic compounds detection

A 2.0 g portion of each sample was placed in a 20 mL headspace vial and sealed with a magnetic screw cap. After incubation for 30 min, 200 μL headspace was injected into the GC-IMS injection port (80 °C) through the injection needle (above 80 °C). High-purity nitrogen (N₂, purity ≥99.999%) was used as the carrier gas. The carrier-gas flow program was as follows: 2 mL/min for 2 min, increased to 15 mL/min over 8 min, then to 100 mL/min over 10 min, and finally to 150 mL/min over the last 10 min. The total running time of GC was 30 min. The drift gas temperature and flow rate (N_2_, purity ≥99.999%) were 45 °C and 150 mL/min, respectively. The instrument was operated in positive ion mode. Each spectrum was acquired with an average of 12 scans.

To normalize retention indices, each standard solution (n-ketone C4-C9) was diluted to 0.8 g/mL. Subsequently, the stock solutions of each standard were mixed in a 1:1:1:1:1:1 volume ratio and diluted with ultrapure water to 1000 mg/L. The mixed standard stock solution was stored at 4 °C. Before analysis, the stock solution was diluted with ultrapure water to obtain a working concentration of 200 mg/L for each standard. Then, add 1 mL of the solution to a 20 mL headspace vial and perform detection using the optimized method. For repeatability evaluation, a fresh 200 mg/L working solution containing 2-pentanone was prepared daily by diluting the mixed standard stock solution with ultrapure water.

### 2.9 Sensory evaluation

Sensory evaluation was conducted according to the evaluation procedure provided by Shuangyu Food Co., Ltd. The pork jerky samples were evaluated for color, aroma, texture and aftertaste, and overall acceptability using a 9-point scoring scale. Detailed panel procedures and scoring criteria are provided in Supplementary Method S1 and Table S1.

### 2.10 Chemometrics methods

A total of 180 sample units from the RAW control and the five drying-time groups were used for chemometric analysis. The peak volumes of the 53 GC-IMS VOC signals were assembled as the predictor matrix X, while moisture content, absorbance at 420 nm (A_420_), fluorescence intensity, and the L*, a*, and b* values were used as response variables Y. An independent regression model was established for each physicochemical index. Because GC-IMS peak volumes represent semi-quantitative signal intensities rather than absolute VOCs concentrations, the resulting models were interpreted as prediction models based on GC-IMS VOC fingerprints.

The Kennard-Stone algorithm was applied to the VOCs feature space to obtain representative subsets with broad coverage of the sample distribution. The 180 samples were divided into a training set, validation set, and test set containing 112, 28, and 40 samples, respectively. The training set was used for model fitting, the validation set for hyperparameter optimization and model selection, and the test set exclusively for final performance evaluation. The VOCs matrix was mean-centered and auto scaled. To prevent information leakage, the centering and scaling parameters were calculated exclusively from the training set and then applied unchanged to the validation and test sets, following the same preprocessing strategy used in the reference study.

Support vector regression (SVR) was used to model the potentially nonlinear relationships between the GC-IMS VOC fingerprints and the physicochemical indices. The SVR regression function is expressed as (Smola & Schölkopf, 2004).

(2) $$f\left(x\right)=W^{T}\phi \left(X\right)+b$$

where x is the VOCs feature vector, $\phi \left(x\right)$ maps the original variables into a feature space, w is the weight vector, and b is the intercept. SVR minimizes model complexity and prediction error using the ε-insensitive loss function:

(3) $$\min_{W,b}\left[\frac{1}{2}{\left\|w\right\|}^{2}+C\sum_{i=1}^{n}L_{\varepsilon }(y_{i},f\left(x_{i}\right))\right],L_{\varepsilon }=\max\left(0,\left|y_{i},f\left(x_{i}\right)\right|-\varepsilon \right)$$

where C controls the balance between model complexity and prediction error, and εdefines the error-insensitive region. Through kernel mapping, SVR can describe nonlinear relationships without explicitly constructing high-dimensional transformed variables. Its regularized formulation is particularly suitable for GC-IMS data containing numerous correlated VOC signals and a moderate number of samples.

Extra trees regression (ETR) was employed as a complementary tree-based ensemble algorithm. ETR constructs multiple regression trees by randomly selecting candidate variables and split thresholds, and the final prediction is obtained by averaging the outputs of all trees (Geurts et al., 2006):

(4) $$\hat{y}\left(X\right)=\frac{1}{T}\sum_{i=1}^{T}h_{t}\left(X\right)$$

where T is the number of trees and $h_{t}\left(x\right)$ is the prediction generated by the tth tree. The extensive randomization of ETR reduces the instability of individual decision trees and enables the model to capture nonlinear responses, threshold effects, and interactions among VOC variables. SVR and ETR were selected because they represent complementary nonlinear modeling mechanisms. SVR estimates a regularized and relatively smooth global relationship in a kernel-defined feature space, whereas ETR describes local and interaction-dependent relationships through randomized partitions of the predictor space. Their comparison therefore allowed the predictive information contained in the GC-IMS VOC fingerprints to be evaluated across different model structures rather than relying on a single algorithm. The principal hyperparameters of both models were optimized according to validation-set performance.

SHapley Additive exPlanations (SHAP) was applied to interpret the SVR and ETR models and quantify the contribution of each VOC variable to the predicted physicochemical indices. For an individual sample, the model output was represented as (Lundberg & Lee, 2017)

(5) $$f\left(x\right)=\phi_{0}+\sum_{j=1}^{M}\phi_{j}$$

where $\phi_{0}$ is the expected model output, M is the number of VOC variables, and $\phi_{j}$ is the SHAP value of the jth variable. Global feature importance was evaluated using the mean absolute SHAP value (Lundberg et al., 2020):

(6) $$I_{j}=\frac{1}{n}\sum_{i=1}^{n}\left|\phi_{\mathrm{ij}}\right|$$

where $\phi_{\mathrm{ij}}$ is the SHAP value of variable j for sample i. A larger mean absolute SHAP value indicated a greater overall contribution to model prediction. The sign and distribution of the SHAP values, together with the corresponding feature values, were used to evaluate whether high or low VOC signals were positively or negatively associated with the predicted physicochemical index. Comparison of the SVR-SHAP and ETR-SHAP results was further used to distinguish consistently important VOCs from model-specific features. To examine drying-stage-dependent variations in local feature attribution, the sample-level SHAP values of the top 10 VOCs for each physicochemical index were grouped according to the RAW, 4, 4.5, 5, 5.5, and 6 h treatments, and the mean signed SHAP value was calculated within each group. For visualization in the supplementary heatmaps, the six group-mean SHAP values of each VOC were standardized by row using *Z*-score normalization.

### 2.11 Model evaluation

The performance of the regression models was evaluated using the coefficient of determination (R^2^), root mean square error (RMSE), and mean absolute error (MAE).

(7) $$R^{2}=1-\frac{\sum_{i=1}^{n}{\left(y_{i,\mathrm{measured}}-y_{i,\mathrm{predicted}}\right)}^{2}}{\sum_{i=1}^{n}{\left(y_{i,\mathrm{measured}}-\bar{y_{\mathrm{measured}}}\right)}^{2}}$$

(8) $$\mathrm{RMSE}=\sqrt{\frac{\sum_{i=1}^{n}\left(y_{i,\mathrm{measured}}-y_{i,\mathrm{predicted}}\right)}{n}}$$

(9) $$\mathrm{MAE}=\frac{\sum_{i=1}^{n}\left|y_{i,\mathrm{measured}}-y_{i,\mathrm{predicted}}\right|}{n}$$

where n is the number of samples, $y_{i,\mathrm{measured}}$ is the measured value of the response variable for the ith sample, $\bar{y_{\mathrm{measured}}}$ is mean measured value of the response variable, and $y_{i,\mathrm{predicted}}$ the corresponding predicted value.

### 2.12 Data analysis and graphing

GC-IMS data were visualized using Vocal software; 2D and 3D spectra were compared using the Reporter plug-in, and visual fingerprints were generated through the Gallery plug-in. Qualitative identification of VOCs was performed using the National Institute of Standards and Technology (NIST) database and the IMS database.

For physicochemical indicators (e.g., moisture, color, fluorescence intensity), statistical analyses were conducted using a mixed linear model (LMM) in SPSS 23. Time was treated as a fixed effect, whereas days and batches were included as random effects in the model. Batches were nested within days to account for potential variability among independent replicate groups. A *P* < 0.05 was considered statistically significant. The model specification for this experimental design, in which days and batches nested within days were treated as random effects, is given as follows:

(10) $$Y_{t,d,b}=\mu +\alpha_{t}+D_{h}+B_{\mathrm{hk}}+\varepsilon_{t,d,b}$$

where $Y_{t,d,b}$ represents the value of the dependent variable measured at time, day, and batch. μ denotes the intercept (the baseline level of the model). $\alpha_{t}$ indicates the fixed effect (the effect of drying time). $D_{h}$ signifies the random effect (the effect of days). $B_{\mathrm{hk}}$ represents the random effect (the effect of batches within days). $\varepsilon_{t,d,b}$ denotes the residual.

Origin 2022 was used to generate line plots and bar charts, while Jupyter Notebook was used for data processing, SHAP value calculation, and the development and evaluation of the SVR and ETR models.

## 3 Results and discussion

### 3.1 Preliminary drying characteristics and moisture evolution of pork jerky

As shown in Table S2, drying time exerted a highly significant effect on the moisture content of pork jerky (F = 1874.057, *P* < 0.001). The estimated variance components for procurement day and batch nested within day were both at the boundary of zero in the fitted mixed model, whereas the residual variance was 6.471. Thus, within the present dataset, no additional between-day or within-day batch variance was detected beyond the residual variability. Moisture content is one of the most critical quality parameters of dried meat products, as it directly influences water activity (aw), microbial stability, texture, and shelf life (Ribeiro et al., 2025). Fig. 1 presents the preliminary drying curves obtained over a broad temperature range. These preliminary trials were used to describe the general moisture-loss behavior of pork jerky under different drying temperatures and were not part of the formal physicochemical, free amino acid, VOC, sensory, or modeling analyses. At all investigated temperatures, moisture content decreased progressively with drying time, reflecting continuous moisture evaporation and internal water migration (Chang et al., 2011). Rapid moisture loss was observed during the initial stage of drying, followed by a progressively slower decline as drying proceeded, which is consistent with previous observations for dried meat products (Mediani et al., 2022; Shi, Feng, et al., 2021; Shi, Zhao, et al., 2021).

**Fig. 1:**
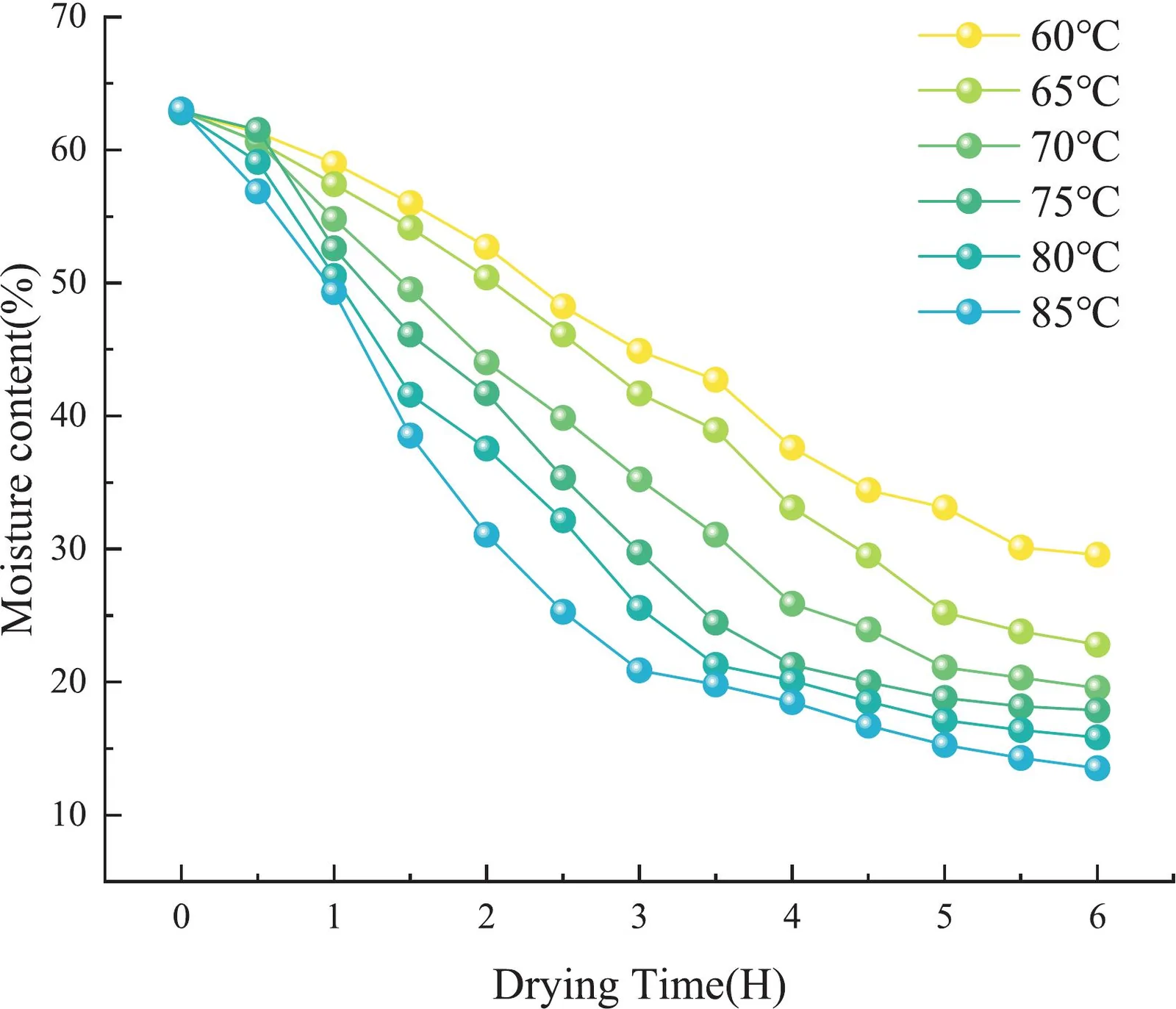
Preliminary drying curves of pork jerky at different drying temperatures.

The formal experiment was designed to investigate quality evolution during the later stage of drying at a fixed temperature of 80 °C. Accordingly, detailed physicochemical, free amino acid, VOC, sensory, and modeling analyses were concentrated within the 4–6 h drying interval, with the marinated undried sample included as the RAW control. Therefore, the present study focuses on quality changes within this selected later drying stage rather than providing a complete kinetic characterization of chemical transformations throughout the entire drying process.

### 3.2 Effect of drying time on color of pork jerky

Color is a crucial sensory attribute of pork jerky, as it directly influences consumer acceptance and perceived quality. The International Commission on Illumination (CIE) has proposed several color representation systems, including the CIE-RGB color space, CIE-XYZ, and CIE L*a*b* systems. Among these, the CIE L*a*b* color model is designed to approximately encompass all colors perceptible to the human eye. The model consists of a lightness component (L*) and two chromaticity coordinates (a* and b*), where L* represents lightness, positive a* values indicate red and negative values indicate green, while positive b* values represent yellow and negative values represent blue. This model was recommended by the Meat Color Evaluation Guidelines Committee of the American Meat Science Association as the standard method for meat color evaluation (King et al., 2023). Therefore, the CIE L*a*b* color model was employed in this study to evaluate the color characteristics of dried meat products.

As shown in Tables S3-S5, drying time had a highly significant effect on color parameters (*P* < 0.001), whereas the effects of date and batch on color variation were negligible. As shown in Fig. 2, the L* value increased steadily with prolonged drying time. Compared with RAW samples (L* = 54.35), samples dried for 5 h or longer exhibited pronounced increases in L* values (64.97–66.03). This increase in lightness is likely attributable to reduced surface moisture, which decreases light absorption and enhances light scattering (Ruedt et al., 2023). Additionally, thermal denaturation of myofibrillar proteins may further increase surface reflectance, contributing to the brighter appearance (Ruedt et al., 2023). The a* value exhibited a continuous decline with increasing drying time, decreasing from 14.27 in RAW samples to 8.44 after 6 h. The reduction in redness is consistent with thermal degradation and oxidation of myoglobin, processes that are accelerated under low-moisture and high-temperature conditions. During prolonged drying (≥5 h), pigment oxidation became more pronounced, resulting in a darker brownish appearance and reduced red intensity. This trend is consistent with color changes commonly reported in heat-treated meat products (Álvarez et al., 2023). In contrast to a*, the b* value increased with extended drying time, rising from 21.18 in RAW samples to 27.71 after 6 h. The enhancement of yellowness is characteristic of the formation of Maillard reaction intermediates and advanced melanoidins, which shift color toward yellow-brown hues (Hu et al., 2024). The most pronounced increase occurred after 4.5 h, corresponding to the onset of intensified non-enzymatic browning as moisture levels decreased and reactants became more concentrated. This observation is consistent with the observed increases in fluorescence and absorbance indices associated with Maillard-derived compounds.

**Fig. 2:**
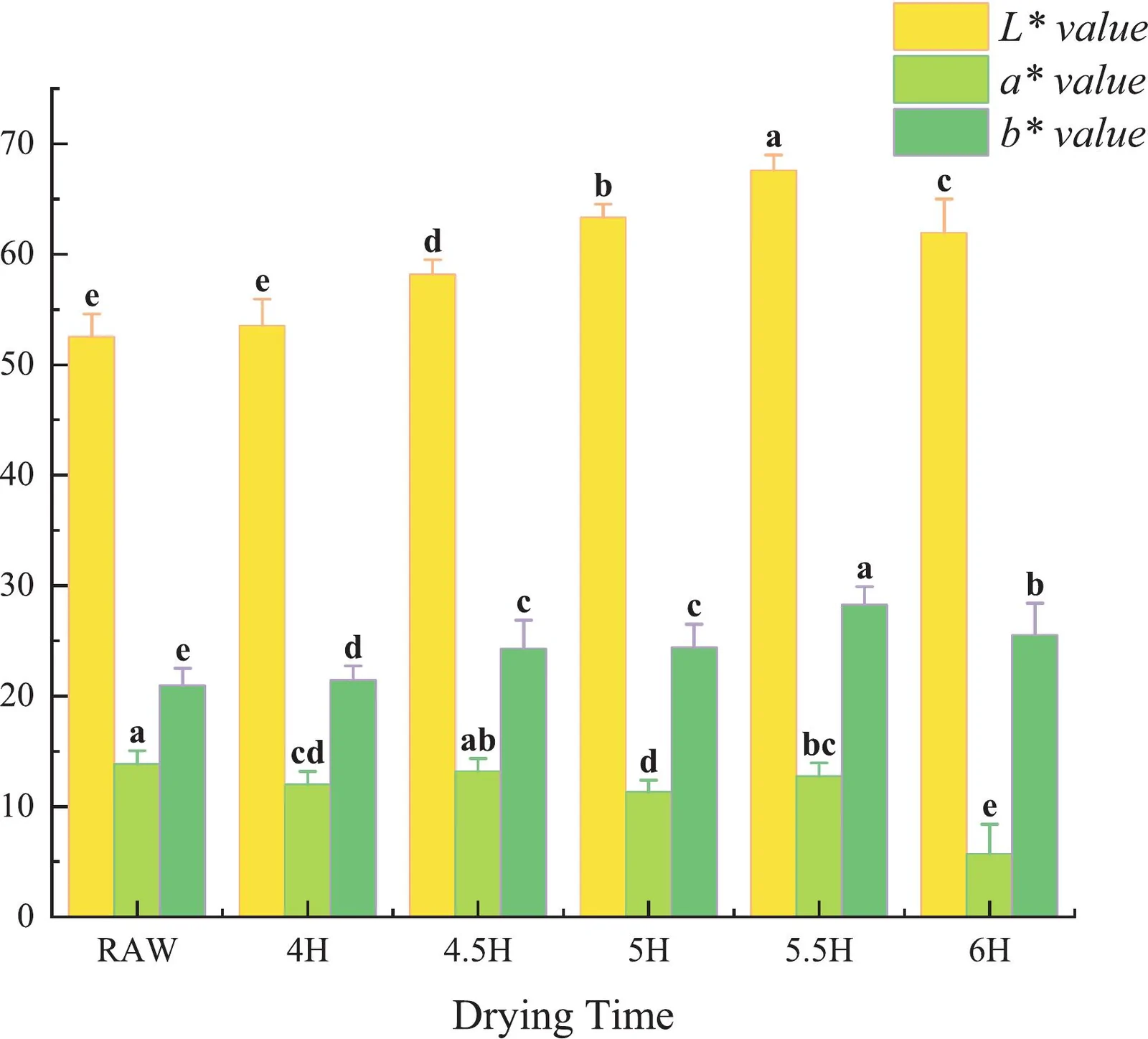
L*, a* and b* values of pork jerky at different drying times. Different lowercase letters indicate significant differences among drying times (*P* < 0.05).

Collectively, the observed color changes (higher L* and b* values and lower a* values) indicate that pork jerky became progressively lighter, less red, and more yellow with increasing drying time at 80 °C. Notably, pronounced color changes occurred between 4 and 5.5 h, coinciding with continued moisture reduction and the progressive approach to the final low-moisture state. During this later drying stage, reduced moisture and increasingly concentrated reactants also provided conditions favorable for intensified browning and associated chemical transformations. These results demonstrate pronounced quality development within the selected 4–6 h drying interval.

### 3.3 Effect of drying time on Maillard reaction intensity of pork jerky

The degree of the Maillard reaction was evaluated based on the fluorescence intensity and browning level of the pork jerky. Absorbance at 420 nm (A_420_) and fluorescence intensity measured at an excitation wavelength of 370 nm and an emission wavelength of 440 nm were used as indicators to assess browning development. As shown in Tables S6-S7, drying time significantly affected the extent of the Maillard reaction (*P* < 0.001). As shown in Fig. 3, absorbance at 420 nm was lowest in RAW samples (16.82) and increased markedly after 4 h of drying (48.76). It remained at a relatively high level at 4.5 and 5 h (45.71 and 48.92, respectively), reached a maximum at 5.5 h (67.80), and remained similarly high at 6 h (66.03), indicating pronounced browning development during the later drying stage. The high A420 values observed at 5.5 and 6 h coincided with the low-moisture conditions reached during the later drying stage, which may favor browning reactions (Kim et al., 2022).

**Fig. 3:**
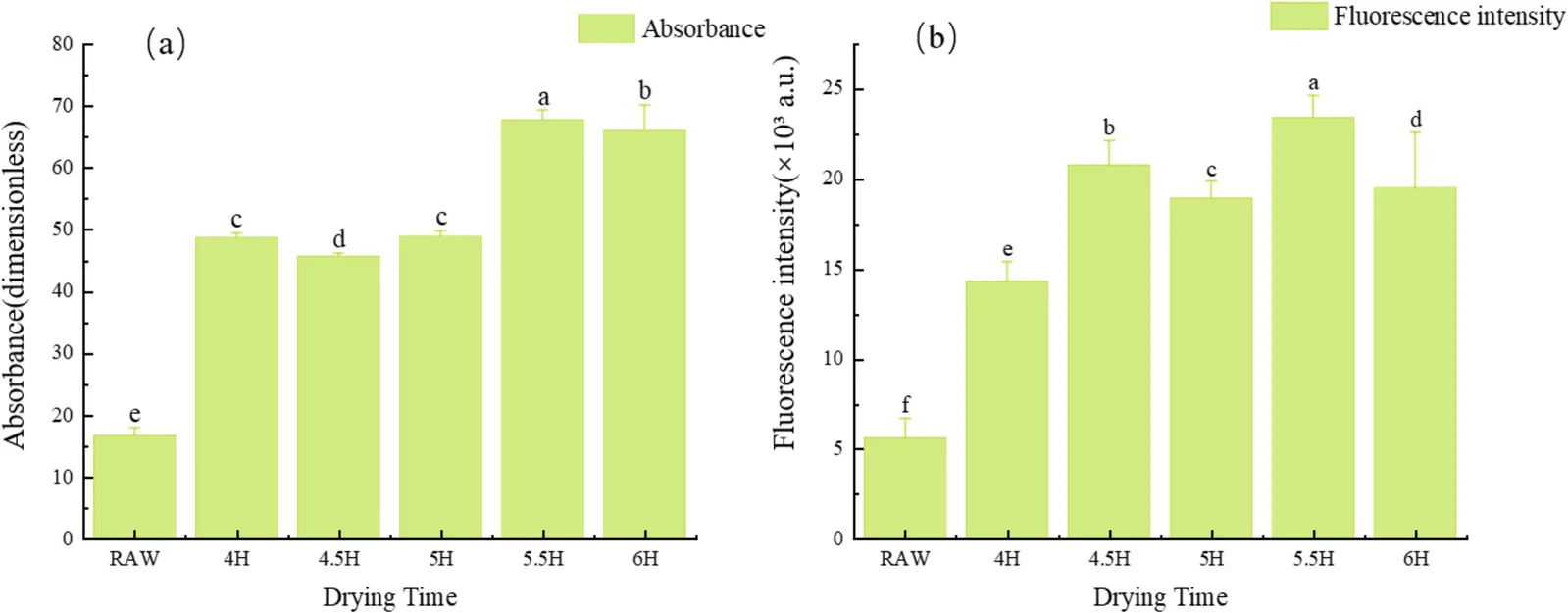
Influence of drying time on Maillard reaction degree. (a) Absorbance, (b) Fluorescence intensity. Mean values within a single trait bearing different lowercase superscript letters (a, b, c) are significantly different (*P* < 0.05).

The compounds detected at Ex 370 nm and Em 440 nm are primarily intermediate reactive products of the Maillard reaction, including reduced ketones and dehydro-reduced ketones. Fluorescence intensity reflects the reaction “activity” or progression potential of the Maillard reaction. These fluorescent intermediates serve as sensitive early markers of Maillard reaction progression (Li et al., 2024). Fluorescence intensity was 5.97 × 10^3^ a.u. in RAW samples and increased to 14.36 × 10^3^ a.u. after 4 h of drying. It further increased during prolonged drying, reaching 23.43 × 10^3^ and 27.96 × 10^3^ a.u. at 5.5 and 6 h, respectively. This trend indicates the progressive accumulation of fluorescent Maillard reaction products during drying. Pearson correlation analysis further revealed significant associations among moisture content, Maillard related indicators, and color parameters (Fig. S1). In particular, moisture content was negatively correlated with A_420_ and fluorescence intensity, whereas A_420_ and fluorescence intensity were positively associated with several color parameters, indicating coordinated physicochemical changes during drying.

### 3.4 Effect of drying time on free amino acids (FAAs) of pork jerky

Free amino acids (FAAs) play a pivotal role in the flavor development of dried meat products (You et al., 2024), acting both as direct taste-active compounds (umami, sweet, and bitter) and as key precursors for Strecker degradation and Maillard reaction-derived volatile compounds during thermal processing. Changes in individual FAA contents (mg/100 g, dry basis) in pork jerky during drying at 80 °C are presented in Fig. 4 and Table S8.

**Fig. 4:**
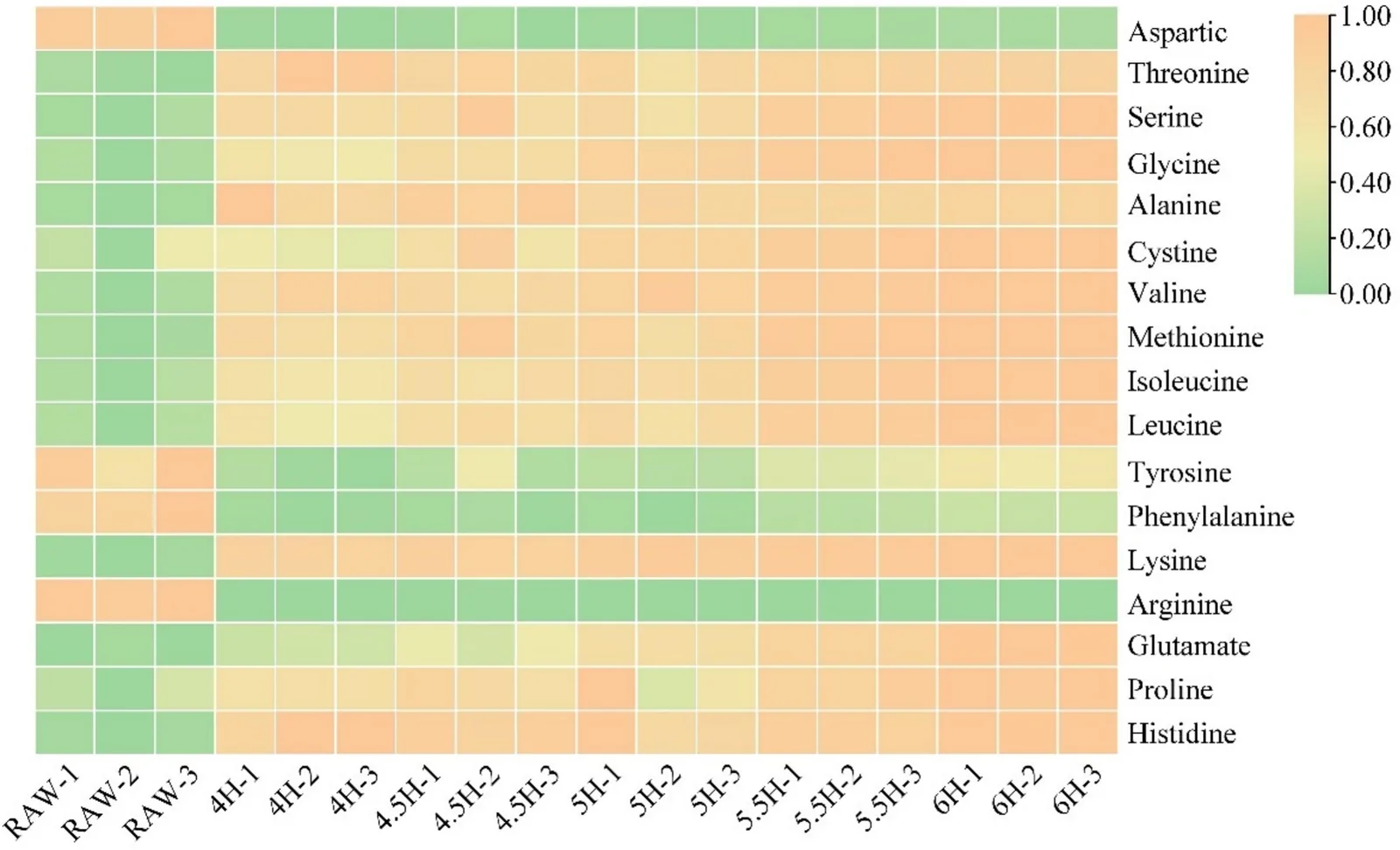
Influence of drying time on the content of free amino acids in pork jerky.

Most FAAs increased significantly (*P* < 0.05) with prolonged drying time. Glutamic acid, the dominant contributor to umami taste (Rotola-Pukkila et al., 2015), increased steadily from 836.82 mg/100 g (dry basis) in RAW samples to 1286.204 mg/100 g after 6 h (+53.70%). In contrast, lysine exhibited the most dramatic increase (>12-fold, from 6.017 to 80.912 mg/100 g), indicating an increased availability of this reactive amino acid for potential participation in late-stage Maillard reactions. Branched-chain amino acids (leucine, valine, and isoleucine) and sweet-tasting amino acids (threonine, serine, glycine, and alanine) also accumulated consistently (Wong et al., 2008). For example, leucine increased from 43.237 to 72.535 mg/100 g, while alanine rose from 88.055 to 157.067 mg/100 g, potentially contributing to taste characteristics while also providing precursors for Strecker aldehyde formation.

In contrast, several amino acids exhibited marked decreases during drying. Arginine decreased precipitously from 443.458 mg/100 g to approximately 19.694 mg/100 g within the first 4 h and remained at low levels thereafter, suggesting substantial changes in its abundance during the early drying stage. Phenylalanine and tyrosine decreased by approximately 33.94% and 27.63%, respectively, which may be associated with their participation in Strecker degradation and other thermal reactions during drying. Histidine exhibited a transient increase at 4 h, likely resulting from carnosine hydrolysis, followed by slight consumption at extended drying times.

Notably, aspartic acid and arginine exhibited extremely high concentrations in RAW samples but declined sharply during drying. This pattern is primarily attributable to the addition of seasonings such as fish sauce, a fermented condiment produced by the enzymatic hydrolysis of fish or shrimp in the presence of salt, which inherently contains high concentrations of arginine, aspartic acid, glutamic acid, and other free amino acids. During pork jerky drying, these free amino acids can serve as substrates for flavor-forming reactions, including the Maillard reaction, which may partly contribute to the observed changes in their contents.

### 3.5 Effect of drying time on VOCs of pork jerky

During the drying process of pork jerky, changes in moisture content and free amino acids were accompanied by continuous alterations in VOCs. A total of 53 volatile compounds were identified across six sample groups, comprising 19 aldehydes, 12 alcohols, 7 ketones, 2 acids, 4 esters, 8 heterocyclic compounds, and 1 miscellaneous compound. The visual fingerprint spectrum generated using the Gallery plugin is shown in Fig. 5, and detailed compound information is provided in Table S9.

**Fig. 5:**
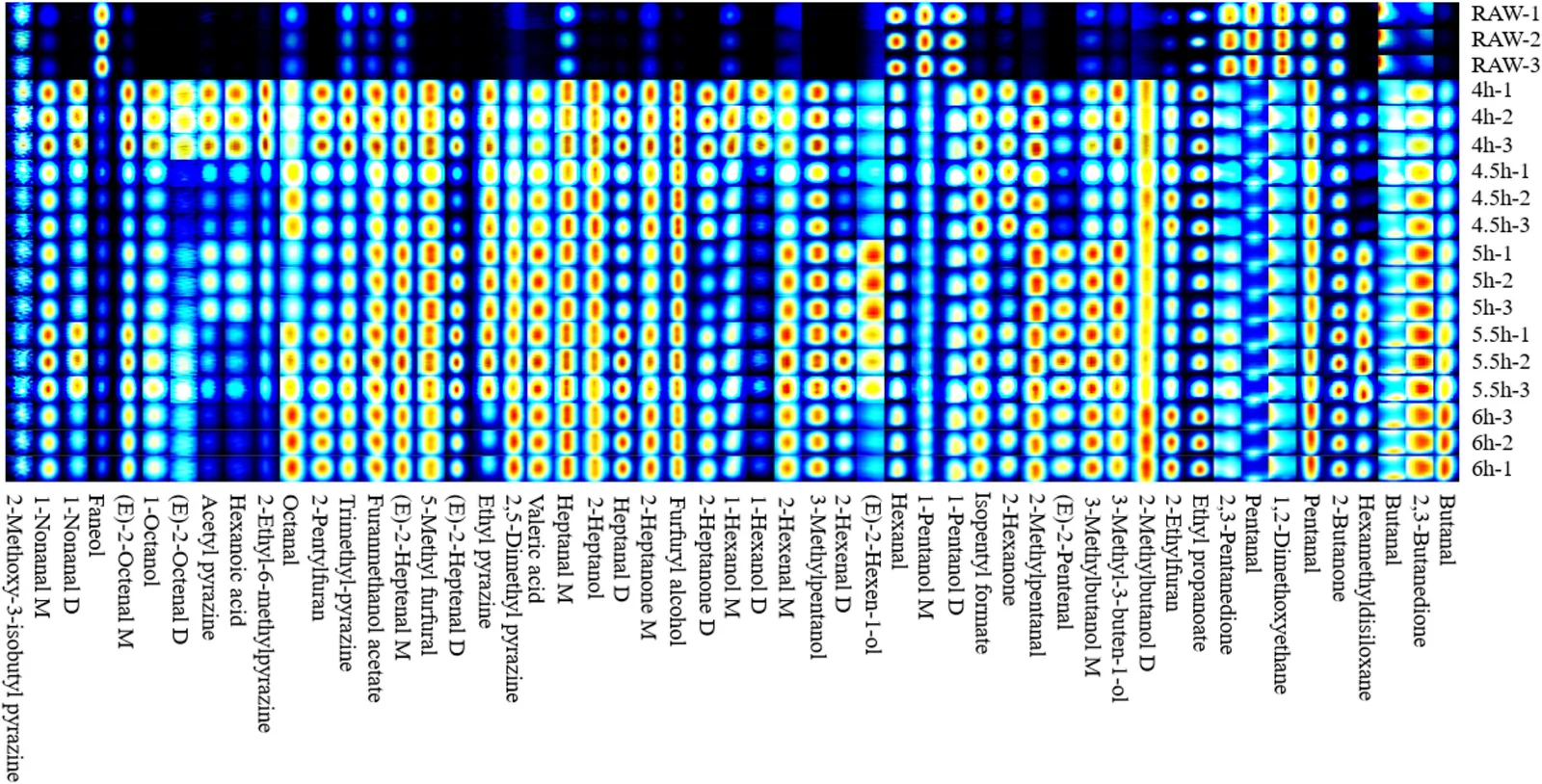
Fingerprinting of VOCs of pork jerky. (D: represents the dimer of the substance; M: represents the monomer of the substance.)

In RAW samples, furaneol, 1-pentanol, and 2,3-pentanedione exhibited relatively high initial GC-IMS signals. Furaneol decreased markedly from 759.191 in RAW samples to 265.482 after 4 h of drying and remained at substantially lower levels during the subsequent drying period. The signals of 1-pentanol and 2,3-pentanedione also generally remained below their RAW levels after drying. In contrast, hexanal showed a fluctuating pattern and reached a signal slightly higher than that of the RAW sample at 6 h, indicating that individual VOCs followed distinct temporal trajectories rather than a uniform decreasing trend.

During drying at 80 °C, several VOCs exhibited pronounced increases after the onset of thermal drying, followed by stage-dependent fluctuations or decreases during the 4–6 h period. For example, the GC-IMS signal of (E)-2-octenal M increased markedly from the RAW group to 4 h and subsequently fluctuated during prolonged drying. A similar non-monotonic pattern was observed for 2-hexanone. The pyrazines 2-ethyl-6-methylpyrazine and ethyl pyrazine also increased sharply after 4 h and then generally decreased during the later drying period. These results indicate that VOC evolution reflected the combined effects of formation, volatilization, and further chemical transformation rather than a simple monotonic increase or decrease.

Some VOCs showed overall increases during drying, although their monomer and dimer signals did not necessarily follow the same temporal pattern. Butanal D increased progressively from 35.994 in RAW samples to 541.116 at 6 h, whereas Butanal M fluctuated during drying. Similarly, Pentanal D generally increased and reached 938.163 at 6 h, while Pentanal M decreased markedly after drying. The signal of 2,3-butanedione increased from 59.633 in RAW samples to 212.482 at 5.5 h before a slight decline at 6 h. Ethyl propanoate remained substantially higher in all dried samples than in RAW samples and reached its highest signal at 6 h. These compound-specific patterns further demonstrate that VOC evolution during drying reflects concurrent formation, transformation, volatilization, and loss. Collectively, these changes may contribute to the evolving aroma profile of pork jerky during drying.

Heptanal, 2-heptanone, and furfuryl alcohol showed substantial increases after thermal drying compared with the RAW samples, followed by comparatively smaller fluctuations during the 4–6 h interval. For example, Heptanal M increased from 234.757 in RAW samples to 594.631 at 4 h and subsequently remained within a relatively narrow range of 569.683–627.476. Furfuryl alcohol similarly increased from 55.543 in RAW samples to 807.797 at 4 h and remained at relatively high levels thereafter. Thus, these compounds are better described as being strongly elevated after drying and comparatively stable or fluctuating within the selected later drying window, rather than remaining stable throughout the entire process.

### 3.6 Sensory evaluation

Drying time significantly affected all sensory attributes of pork jerky (*P* < 0.001; Table 1 and Fig. 6). The scores for color, texture and aftertaste, and overall acceptability increased from 4 to 5 h and subsequently declined, with the 5 h samples showing the highest scores of 8.53 ± 0.57, 8.63 ± 0.49, and 8.43 ± 0.57, respectively. Aroma showed a similar overall trend, although no significant difference was observed between 5 and 5.5 h. The improved sensory quality up to 5 h coincided with progressive moisture removal, color development, Maillard reactions, and substantial changes in the VOCs profile. After 5 h, further drying resulted in lower color, texture, and overall acceptability scores, despite the relatively high aroma score at 5.5 h. This may reflect the continued formation or accumulation of aroma-related volatile compounds during the later drying stage, whereas further dehydration and intensified browning adversely affected texture and appearance. Overall, drying for 5 h provided the most favorable balance among color, aroma, texture, and overall acceptability.

**Table 1:** Sensory evaluation scores of pork jerky subjected to different drying times.

Drying time	4H	4.5H	5H	5.5H	6H	*P*
Color	5.77 ± 0.57e	6.83 ± 0.65c	8.53 ± 0.57a	7.33 ± 0.66b	6.20 ± 0.55d	<0.001
Aroma	5.40 ± 0.72d	6.50 ± 0.51c	8.27 ± 0.58a	7.87 ± 0.78a	7.00 ± 0.59b	<0.001
Texture	5.63 ± 0.72c	6.70 ± 0.53b	8.63 ± 0.49a	7.0 ± 0.64b	5.70 ± 0.60c	<0.001
Overall	5.37 ± 0.56e	6.63 ± 0.49c	8.43 ± 0.57a	7.17 ± 0.53b	6.03 ± 0.41d	<0.001

Notes: Values are expressed as mean ± SD. Different lowercase superscript letters within the same row indicate significant differences among drying times (*P* < 0.05).

**Fig. 6:**
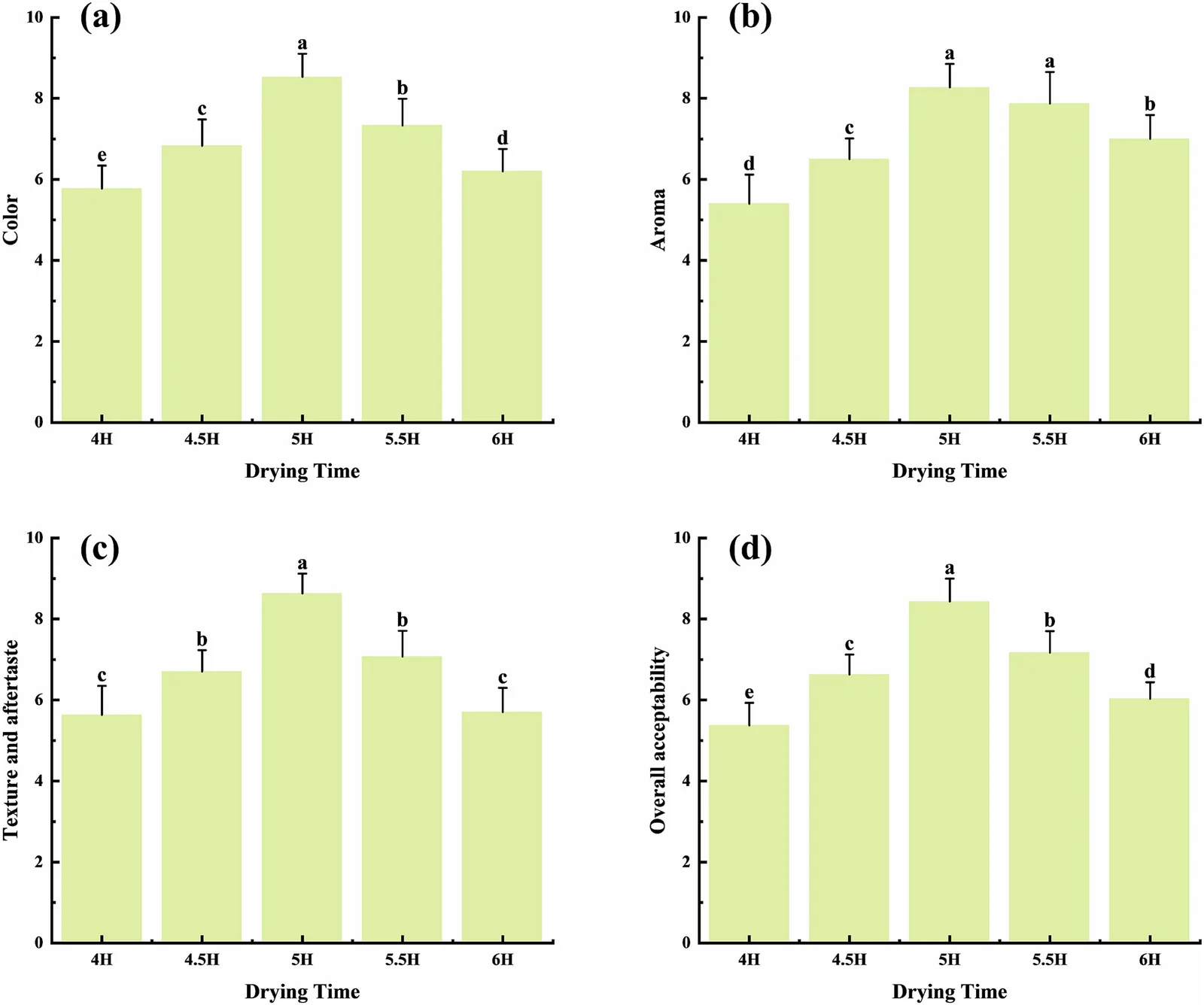
Effects of drying time on the sensory attributes of pork jerky. (a) Color, (b) aroma, (c) texture and aftertaste, and (d) overall acceptability. Values are expressed as mean ± SD. Different lowercase letters indicate significant differences among drying times (*P* < 0.05).

### 3.7 Evaluation of the predictive performance of SVR and ETR models based on GC-IMS VOC fingerprints for the physicochemical indices of pork jerky

Drying-induced moisture loss, lipid oxidation, Maillard reaction, Strecker degradation, and color development occurred concurrently with changes in the VOCs profiles of pork jerky. Therefore, the GC-IMS VOC fingerprints were used to predict moisture content, absorbance at 420 nm, fluorescence intensity, and the L*, a*, and b* values using SVR and ETR models. As shown in Fig. 7 and Table 2, both models provided high predictive performance, and the measured and predicted values were distributed close to the ideal 1:1 line. The similar results obtained for the training, validation, and test sets indicated that neither model showed obvious overfitting. For SVR, the test-set R^2^ values ranged from 0.996 to 0.998. Specifically, the R^2^ values for moisture content, absorbance, fluorescence intensity, L*, a*, and b* were 0.998, 0.997, 0.996, 0.997, 0.997, and 0.997, respectively. The corresponding RMSE values were 0.724, 0.602, 0.233, 0.224, 0.123, and 0.161.

**Fig. 7:**
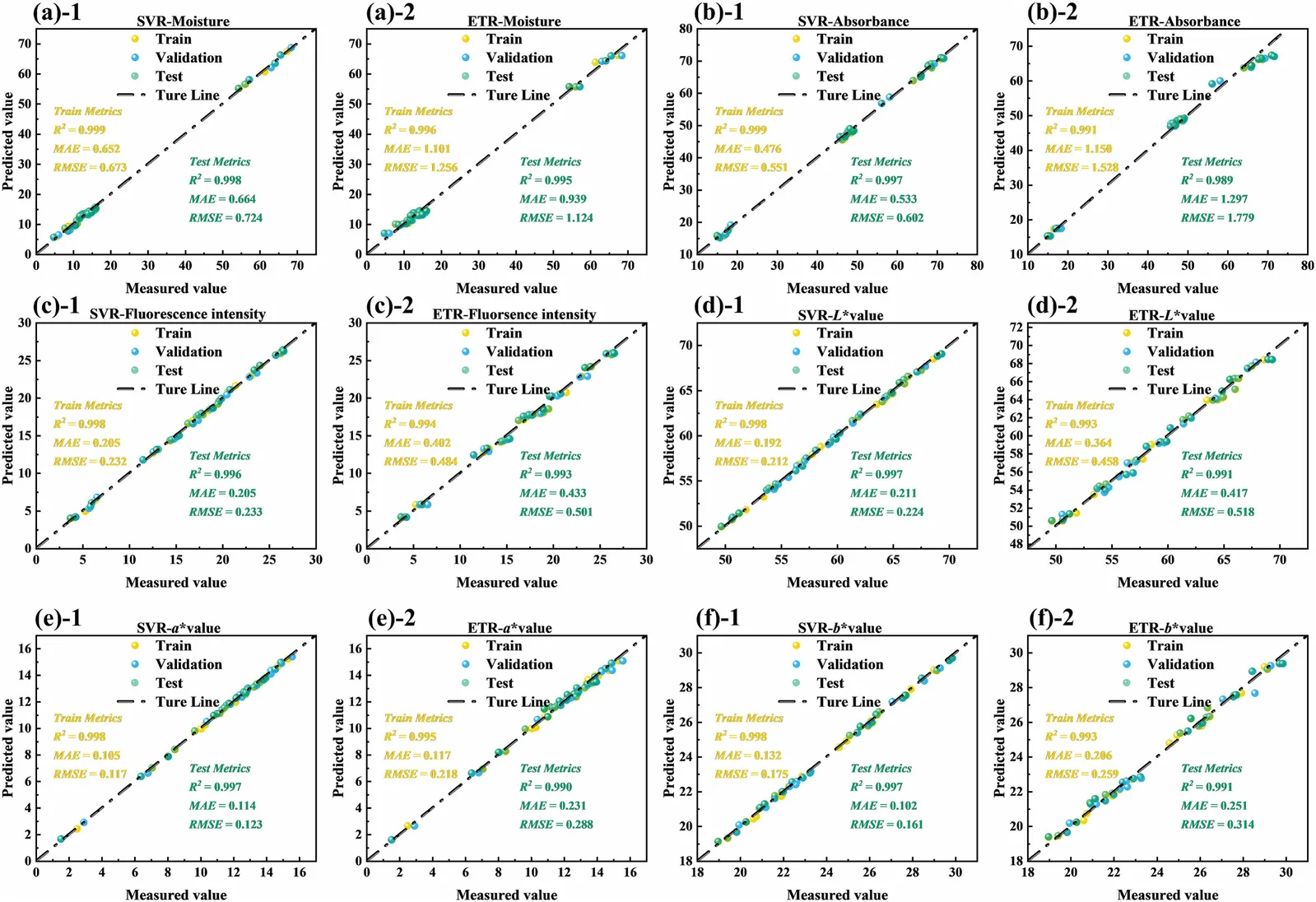
Measured versus predicted values of the physicochemical indices of pork jerky obtained using SVR and ETR models based on GC-IMS VOC fingerprints. Panels (a-f)-1 show the SVR results for moisture content, absorbance at 420 nm, fluorescence intensity, L*, a*, and b* values, respectively. Panels (a-f)-2 show the corresponding ETR results for the same indices. The training, validation, and test samples are distinguished by different colors, and the dashed line represents the ideal 1:1 relationship between the measured and predicted values.

**Table 2:** Predictive performance of SVR and ETR models for the physicochemical indices of pork jerky based on GC-IMS VOC fingerprints.

Indicator	Train set			Validation set			Test set		
	R^2^	MAE	RMSE	R^2^	MAE	RMSE	R^2^	MAE	RMSE
*SVR*									
Moisture	0.999	0.625	0.673	0.998	0.628	0.678	0.998	0.664	0.724
Absorbance	0.999	0.476	0.551	0.997	0.539	0.601	0.997	0.533	0.602
Fluorescence intensity	0.998	0.205	0.232	0.997	0.218	0.243	0.996	0.205	0.233
L*value	0.998	0.192	0.212	0.997	0.188	0.209	0.997	0.211	0.224
a*value	0.998	0.105	0.117	0.997	0.119	0.129	0.997	0.114	0.123
b*value	0.998	0.132	0.175	0.996	0.131	0.275	0.997	0.102	0.161
*ETR*									
Moisture	0.996	1.101	1.256	0.995	0.983	1.175	0.995	0.939	1.124
Absorbance	0.991	1.150	1.528	0.990	1.706	1.257	0.989	1.297	1.779
Fluorescence intensity	0.994	0.402	0.484	0.994	0.431	0.511	0.993	0.433	0.501
L*value	0.993	0.364	0.458	0.991	0.397	0.489	0.991	0.417	0.518
a*value	0.995	0.117	0.218	0.994	0.198	0.251	0.990	0.231	0.288
b*value	0.993	0.206	0.259	0.990	0.248	0.316	0.991	0.251	0.314

ETR also achieved satisfactory test-set performance, with R^2^ values of 0.995, 0.989, 0.993, 0.991, 0.990, and 0.991 for the six physicochemical indices, respectively. However, its RMSE and MAE values were consistently higher than those of SVR. The difference was particularly evident for absorbance, for which SVR achieved a test-set RMSE of 0.602, compared with 1.779 for ETR. SVR also produced lower test-set RMSE values for moisture content and fluorescence intensity than ETR, at 0.724 versus 1.124 and 0.233 versus 0.501, respectively.

The overall superiority of SVR may be attributed to its regularized kernel-based structure, which is suitable for extracting smooth nonlinear relationships from moderate-sized datasets containing high-dimensional and correlated GC-IMS variables. In contrast, ETR describes nonlinear relationships and variable interactions through randomized local partitions, but its predictions may be more sensitive to the distribution of samples within these partitions. Nevertheless, the strong performance of both algorithms indicated that the GC-IMS VOC fingerprints contained robust information associated with the physicochemical changes occurring during pork jerky drying. These results were demonstrated within the present sample source and drying-time range, and independent production batches would be required to evaluate the external applicability of the models.

### 3.8 SHAP-based identification of key predictive VOCs

SHAP analysis was used to identify the VOCs that contributed most strongly to the prediction of each physicochemical index and to compare the feature-selection patterns of the SVR and ETR models. The SHAP summary plots are presented in Fig. 8, while the top 10 VOCs ranked by mean absolute SHAP value and their relative contributions are listed in Tables S10 and S11. The rankings differed among physicochemical indices, indicating that the prediction of moisture content, Maillard-related indices, and color parameters relied on different components of the GC-IMS VOC fingerprints.

**Fig. 8:**
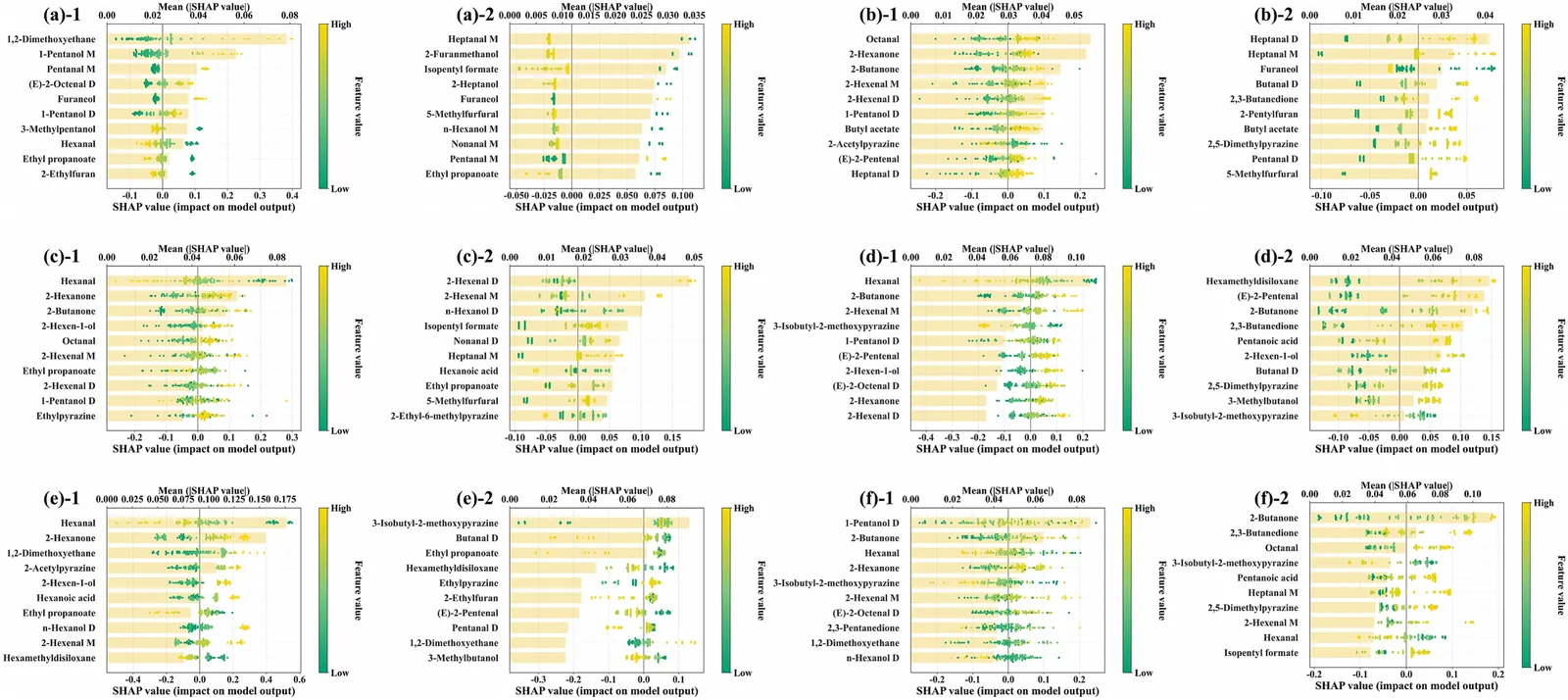
SHAP-based interpretation of SVR and ETR models for the prediction of pork jerky physicochemical indices using GC-IMS VOC fingerprints. Panels (a-f)-1 show the SVR-SHAP results for moisture content, absorbance at 420 nm, fluorescence intensity, L*, a*, and b* values, respectively. Panels (a-f)-2 show the corresponding ETR-SHAP results for the same indices. The horizontal bars represent mean absolute SHAP values, the points represent sample-level SHAP values, and point color indicates the relative feature value from low to high.

For the SVR models, 1,2-dimethoxyethane and 1-pentanol M were the two most important variables for moisture prediction, contributing 19.44% and 13.97%, respectively, among the top 10 features. Octanal and 2-hexanone were dominant for absorbance at 420 nm, whereas hexanal, 2-hexanone, and 2-butanone showed the largest contributions to fluorescence-intensity prediction. Hexanal was also ranked first for the prediction of both L* and a*, while 1-pentanol D and 2-butanone were the leading variables for b*. These results indicate that alcohols, aldehydes, ketones, esters, and heterocyclic compounds jointly contributed predictive information for estimating moisture content, browning-related indices, and color parameters. The ETR-SHAP results produced a partially different feature-ranking pattern. Heptanal M and Furfuryl alcohol were the leading variables for moisture prediction, while heptanal D and heptanal M made the greatest contributions to absorbance prediction. 2-Hexenal D, 2-hexenal M, and n-hexanol D made the largest contributions to fluorescence-intensity prediction. For the color indices, (E)-2-pentenal was highly ranked for L*, 3-isobutyl-2-methoxypyrazine and butanal D were dominant for a*, and 2-butanone and 2,3-butanedione were the most important variables for b*. The SHAP analysis identified several aldehydes and alcohols, compound classes commonly associated with lipid oxidation, together with ketones, furans, and pyrazines, some of which are commonly associated with thermal reactions and Maillard chemistry, as important predictive features. These model-derived associations suggest that the GC-IMS fingerprint contains information related to multiple chemical processes rather than a single VOC class. However, the SHAP results do not demonstrate that these individual VOCs directly participated in, or were causally generated by, these reactions in the present samples.

To further characterize the temporal variation in local feature attribution, drying-time-resolved mean SHAP patterns were examined for both models (Figs. S2 and S3). The results revealed clear stage-dependent changes in the contributions of the major VOC features. For moisture prediction, many important VOCs showed relatively high contributions in the RAW group and lower contributions after drying, whereas the attribution patterns for absorbance, fluorescence intensity, and color parameters varied more strongly across the 4–6 h interval, with pronounced changes in several features around 5 h. These results indicate that the associations captured by the models were dynamic across drying stages and cannot be fully represented by a single global SHAP ranking.

Despite differences in ranking, several VOCs were consistently selected by both algorithms. Pentanal M, furaneol, and ethyl propanoate were shared among the top 10 moisture-related variables. Heptanal D and butyl acetate were common to the absorbance models, while 2-hexenal M, 2-hexenal D, and ethyl propanoate were shared for fluorescence intensity. The common variables for L* included 2-butanone, $\left(E\right)$-2-pentenal, 2-hexen-1-ol, and 3-isobutyl-2-methoxypyrazine, whereas 2-butanone, hexanal, 2-hexenal M, and 3-isobutyl-2-methoxypyrazine were jointly selected for b*. These repeatedly selected VOCs may be considered comparatively robust candidate features because their importance was retained under two different nonlinear modeling structures. Nevertheless, SHAP values describe contributions to model predictions and statistical associations within the present dataset and should not be interpreted as evidence of direct causal effects.

## 4 Conclusion

This study demonstrated that GC-IMS VOC fingerprints combined with nonlinear regression can effectively capture physicochemical changes occurring during the selected later stage of pork jerky drying at 80 °C. The complementary SVR and ETR models showed strong predictive performance, while SHAP analysis identified VOC features that contributed substantially to model predictions of moisture content, Maillard-related indices, and color parameters. Sensory evaluation further indicated that drying for 5 h provided the most favorable overall balance among color, aroma, texture and aftertaste, and acceptability. These results support the potential of integrating GC-IMS fingerprints, physicochemical measurements, sensory evaluation, and interpretable machine learning for characterizing quality evolution during pork jerky processing.

Several limitations should nevertheless be acknowledged. Detailed chemical sampling was concentrated within the 4–6 h drying window, and therefore the present data do not provide a complete kinetic description of chemical changes during the earlier dehydration stage. Although FAA contents were normalized on a dry-matter basis, possible matrix-dependent differences in extraction efficiency cannot be completely excluded. In addition, GC-IMS peak volumes provide semi-quantitative signal responses rather than absolute VOCs concentrations, and the prediction models were evaluated using internal subsets derived from the present raw-material source and processing conditions. Future studies should therefore extend chemical sampling to earlier drying stages, quantitatively validate key VOCs candidates using targeted analytical methods, evaluate model transferability using independent production batches and processing environments, and further integrate chemical markers with sensory responses.

## Ethics statement for sensory evaluation

The sensory evaluation conducted in this study involved trained panelists and did not include any invasive procedures, clinical interventions, or vulnerable populations. Formal ethical approval was not required for this type of sensory evaluation under the applicable institutional and national requirements. All panelists participated voluntarily after being informed of the purpose and procedures of the sensory evaluation. Informed consent was obtained from all participants prior to the evaluation. Participants were free to withdraw from the study at any time, and their privacy and confidentiality were protected throughout the study.

## CRediT authorship contribution statement

**Xinyu Chen:** Writing – review & editing, Supervision, Methodology, Conceptualization. **Haoran Huang:** Writing – review & editing, Writing – original draft, Methodology, Investigation, Formal analysis, Data curation, Conceptualization. **Huangyu Chen:** Writing – review & editing. **Siying Wang:** Writing – review & editing. **Zeyuan Zang:** Writing – review & editing. **Yujia Huo:** Writing – review & editing. **Zhixin Xiong:** Writing – review & editing, Supervision, Methodology, Formal analysis, Conceptualization. **DaoLi Lu:** Writing – review & editing. **Yan Li:** Writing – review & editing.

## Ethics declaration

### Informed consent and patient details

Written informed consent to take part in the study and to publish the article has been obtained from all participants or their legal representatives. The privacy rights of participants have been observed.

### Studies in human

This study was performed in compliance with relevant laws, regulatory frameworks and guidelines where the research took place.

This study was conducted in accordance with Declaration of Helsinki.

This study was approved by the Ethics Review Committee of Nanjing Forestry University. (Approval No. 2026096).

## Declaration of competing interest

The authors declare that they have no known competing financial interests or personal relationships that could have appeared to influence the work reported in this paper.

## Data Availability

Data will be made available on request.
