# Supplementary material for: Effects of drying time on physicochemical properties and volatile organic compounds of pork jerky revealed by GC-IMS and interpretable machine learning
Source: Food Chem X. 2026 Oct 8;39:104562. doi: 10.1016/j.fochx.2026.104562 (PMC13683453; doi:10.1016/j.fochx.2026.104562)
Supplement: Supplementary file 1 [file mmc1.docx]

***Supporting Information for***

Effects of Drying Time on Physicochemical Properties and Volatile Organic Compounds of Pork Jerky Revealed by GC-IMS and Interpretable Machine Learning

Xinyu Chen^a^, Haoran Huang^b^, Huangyu Chen^a^, Siying Wang^a^, Zeyuan Zang^a^, Yujia Huo^c^, Zhixin Xiong ^d,e,^ *, DaoLi Lu^f^, Yan Li^a,^ *

*^a^ School of photoelectric engineering of Changzhou Institute of Technology. Changzhou, Jiangsu 213002, China*

*^b^ College of Chemical Engineering, Xiangtan University, Xiangtan. 411105, China*

*^c^ Shandong Hanon Science Instrument Co., Ltd. Dezhou Shandong 253000, China*

*^d^ College of Light Industry and Food Engineering, Nanjing Forestry University. Nanjing, Jiangsu 210037, China*

*^e^ State Key Laboratory for Development and Utilization of Forest Food Resources, Nanjing Forestry University, Nanjing 210037, China*

*^f^* *School of food and biological engineering, Jiangsu University, Zhenjiang, 212013, China*

*Corresponding author. E-mail address: njfu_xzx@163.com (Zhixin Xiong),

## Table of Contents

| Description | Page |
| --- | --- |
| Supplementary Method S1. Sensory evaluation | S3 |
| Table S1. Sensory evaluation criteria for pork jerky subjected to different drying times. | S3 |
| Fig. S1. Pearson correlation heatmap of physicochemical indicators of pork jerky during drying. | S4 |
| Supplementary analysis of local SHAP values | S5-S6 |
| Fig. S2. Local SHAP patterns of the top 10 VOCs identified by the SVR models for predicting pork jerky physicochemical indices. | S5 |
| Fig. S3. Local SHAP patterns of the top 10 VOCs identified by the ETR models for predicting pork jerky physicochemical indices. | S6 |
| Table S2. Linear mixed model analysis results for drying time on pork jerky moisture content. | S7 |
| Table S3. Linear mixed model analysis results for drying time on pork jerky *L** value. | S8 |
| Table S4. Linear mixed model analysis results for drying time on pork jerky *a** value. | S9 |
| Table S5. Linear mixed model analysis results for drying time on pork jerky *b** value. | S10 |
| Table S6. Linear mixed model analysis results for drying time on pork jerky fluorescence intensity. | S11 |
| Table S7. Linear mixed model analysis results for drying time on pork jerky absorbance. | S12 |
| Table S8. Influence of drying time on the content of free amino acids in pork jerky. (mg/100 g dry weight) | S13-S14 |
| Table S9. Information on volatile organic compounds obtained by GC-IMS. | S15-S17 |
| Table S10. Top 10 VOCs identified by SVR-SHAP for the prediction of pork jerky physicochemical indices. | S18-S20 |
| Table S11. Top 10 VOCs identified by ETR-SHAP for the prediction of pork jerky physicochemical indices. | S21-S23 |

*Supplementary Method S1. Sensory evaluation*

Sensory evaluation was conducted according to the evaluation procedure provided by Shuangyu Food Co., Ltd. A sensory panel consisting of 10 trained panelists (five males and five females) was established. Before evaluation, all panelists were familiarized with the sensory attributes, scoring criteria, and evaluation procedures. Each panelist evaluated each drying treatment three times, resulting in 30 ratings per treatment

The five drying-time treatments (4, 4.5, 5, 5.5, and 6 h at 80 °C) were evaluated for four sensory attributes: color, aroma, texture and aftertaste, and overall acceptability. Each attribute was scored on a 9-point scale, with higher scores representing more desirable sensory characteristics. Scores of 1-3, 4-6, and 7-9 corresponded to low, intermediate, and high sensory quality, respectively, according to the attribute-specific descriptors provided in Table S1.

Table S1. Sensory evaluation criteria for pork jerky subjected to different drying times.

| Index | Sensory descriptors | Score |
| --- | --- | --- |
| Color | Dull yellow appearance with poor gloss | 1-3 |
|  | Reddish-brown color with moderate color intensity | 4-6 |
|  | Bright reddish appearance with good gloss and high color intensity | 7-9 |
| Aroma | Weak characteristic meat aroma with noticeable off-odor | 1-3 |
|  | Slightly weak characteristic meat aroma with slight off-odor | 4-6 |
|  | Intense characteristic meat aroma with no detectable off-odor | 7-9 |
| Texture | Tough and hard texture with little aftertaste | 1-3 |
|  | Slightly tough and dry texture with a pleasant aftertaste | 4-6 |
|  | Tender texture, easy to chew, with a pleasant aftertaste | 7-9 |
| Overall | Low (unacceptable) | 1-3 |
|  | Moderate | 4-6 |
|  | High (highly acceptable) | 7-9 |

Fig. S1. Pearson correlation heatmap of physicochemical indicators of pork jerky during drying. Pearson correlation coefficients (r) were calculated using biological replicate level mean values for moisture content, absorbance at 420 nm (A420), fluorescence intensity, and the L, a, and b* values. Values within the heatmap represent Pearson correlation coefficients. *, *P* < 0.05; **, *P* < 0.01; ***, *P* < 0.001.

Pearson correlation analysis revealed coordinated changes among the physicochemical indicators during pork jerky drying (Fig. S1). Moisture content was strongly negatively correlated with A420 (r = -0.866, *P* < 0.001) and fluorescence intensity (r = -0.788, *P* < 0.001), whereas A420 was positively correlated with fluorescence intensity (r = 0.853, *P* < 0.001) and b* (r = 0.815, *P* < 0.001). Fluorescence intensity was also positively correlated with b* (r = 0.850, *P* < 0.001), and the strongest correlation was observed between L* and b* (r = 0.910, *P* < 0.001). These results indicate that moisture loss, Maillard-related changes, and color development occurred concurrently across the investigated treatments. Because these physicochemical parameters were jointly influenced by drying treatment, the observed correlations reflect statistical associations and do not necessarily indicate direct causal relationships.

*Supplementary analysis of local SHAP values*

To further examine the temporal variation in model attribution, the sample-level SHAP values of the top 10 VOCs were grouped according to drying duration and averaged within each group. As shown in Fig. S2, the SVR models exhibited clear drying-stage-dependent changes in local feature contributions. For moisture prediction, several major VOCs, including 1,2-dimethoxyethane, 1-pentanol M, pentanal M, and furaneol, showed their highest relative contributions in the RAW group and generally lower contributions after drying. In contrast, the attribution patterns for absorbance, fluorescence intensity, and color parameters were more stage dependent, with several VOCs, particularly 2-hexenal M, 2-hexenal D, 2-butanone, and hexanal, showing pronounced changes around the intermediate and later drying stages.

Fig. S2. Local SHAP patterns of the top 10 VOCs identified by the SVR models for predicting pork jerky physicochemical indices. Panels (a-f) correspond to moisture content, absorbance at 420 nm, fluorescence intensity, *L**, *a**, and *b** values, respectively. For visualization, the six group-mean SHAP values of each VOC were standardized by row using Z-score normalization. Red and blue indicate relatively higher and lower standardized mean SHAP contributions, respectively.

Similar stage-dependent behavior was observed for the ETR models (Fig. S3). Most major moisture-related VOCs exhibited relatively high contributions in the RAW group followed by lower contributions after drying, whereas the VOCs associated with absorbance, fluorescence intensity, and color showed more complex temporal patterns. In particular, several features exhibited marked changes around 5 h, including heptanal M and D for absorbance, 2-hexenal M and D for fluorescence intensity, and 2-butanone and related VOCs for the color indices. Overall, the SVR and ETR heatmaps demonstrate that the contribution patterns of important VOCs varied across drying stages rather than remaining constant throughout the process.

Fig. S3. Local SHAP patterns of the top 10 VOCs identified by the ETR models for predicting pork jerky physicochemical indices. Panels (a-f) correspond to moisture content, absorbance at 420 nm, fluorescence intensity, *L**, *a**, and *b** values, respectively. For visualization, the six group-mean SHAP values of each VOC were standardized by row using Z-score normalization. Red and blue indicate relatively higher and lower standardized mean SHAP contributions, respectively.

Table S2. Linear mixed model analysis results for drying time on pork jerky moisture content.

| **Parameters** | **Levels** | **Estimation** | **Standard Error** | ***F* value** | **significance** |
| --- | --- | --- | --- | --- | --- |
| **Fixed effects** | | | | | |
| Intercept | - | 61.173 | 0.464 | - | < 0.001 |
| Drying time | RAW | (For reference) | - | 1874.057 | < 0.001 |
|  | 4H | -46.035 | 0.657 | - | < 0.001 |
|  | 4.5H | -47.425 | 0.657 | - | < 0.001 |
|  | 5H | -50.640 | 0.657 | - | < 0.001 |
|  | 5.5H | -47.520 | 0.657 | - | < 0.001 |
|  | 6H | -52.765 | 0.657 | - | < 0.001 |
| **Random effects** | | | | | |
| **Parameters** | **Variance component** | | | | |
| Day | 0.000 | | | | |
| Day×batches | 0.000 | | | | |
| Residual | 6.471 | | | | |

Table S3. Linear mixed model analysis results for drying time on pork jerky *L** value.

| **Parameters** | **Levels** | **Estimation** | **Standard Error** | ***F* value** | **significance** |
| --- | --- | --- | --- | --- | --- |
| **Fixed effects** | | | | | |
| Intercept | - | 52.528 | 0.369 | - | < 0.001 |
| Drying time | RAW | (For reference) | - | 253.277 | < 0.001 |
|  | 4H | 0.997 | 0.521 | - | 0.058 |
|  | 4.5H | 5.651 | 0.521 | - | < 0.001 |
|  | 5H | 10.776 | 0.521 | - | < 0.001 |
|  | 5.5H | 15.073 | 0.521 | - | < 0.001 |
|  | 6H | 9.428 | 0.521 | - | < 0.001 |
| **Random effects** | | | | | |
| **Parameters** | **Variance component** | | | | |
| Day | 0.000 | | | | |
| Day×batches | 0.000 | | | | |
| Residual | 4.079 | | | | |

Table S4. Linear mixed model analysis results for drying time on pork jerky *a** value.

| **Parameters** | **Levels** | **Estimation** | **Standard Error** | ***F* value** | **significance** |
| --- | --- | --- | --- | --- | --- |
| **Fixed effects** | | | | | |
| Intercept | - | 13.837 | 0.280 | - | < 0.001 |
| Drying time | RAW | (For reference) | - | 111.701 | < 0.001 |
|  | 4H | -1.836 | 0.395 | - | < 0.001 |
|  | 4.5H | -0.647 | 0.395 | - | < 0.001 |
|  | 5H | -2.538 | 0.395 | - | < 0.001 |
|  | 5.5H | -1.108 | 0.395 | - | 0.006 |
|  | 6H | -8.131 | 0.395 | - | < 0.001 |
| **Random effects** | | | | | |
| **Parameters** | **Variance component** | | | | |
| Day | 0.000 | | | | |
| Day×batches | 0.000 | | | | |
| Residual | 2.346 | | | | |

Table S5. Linear mixed model analysis results for drying time on pork jerky *b** value.

| **Parameters** | **Levels** | **Estimation** | **Standard Error** | ***F* value** | **significance** |
| --- | --- | --- | --- | --- | --- |
| **Fixed effects** | | | | | |
| Intercept | - | 20.946 | 0.381 | - | < 0.001 |
| Drying time | RAW | (For reference) | - | 50.162 | < 0.001 |
|  | 4H | 0.512 | 0.539 | - | < 0.001 |
|  | 4.5H | 3.356 | 0.539 | - | < 0.001 |
|  | 5H | 3.449 | 0.539 | - | < 0.001 |
|  | 5.5H | 7.309 | 0.539 | - | < 0.001 |
|  | 6H | 4.589 | 0.539 | - | < 0.001 |
| **Random effects** | | | | | |
| **Parameters** | **Variance component** | | | | |
| Day | 0.000 | | | | |
| Day×batches | 0.000 | | | | |
| Residual | 4.350 | | | | |

Table S6. Linear mixed model analysis results for drying time on pork jerky fluorescence intensity (×10³ a.u.).

| **Parameters** | **Levels** | **Estimation** | **Standard Error** | ***F* value** | **significance** |
| --- | --- | --- | --- | --- | --- |
| **Fixed effects** | | | | | |
| Intercept | - | 5.130 | 0.298 | - | < 0.001 |
| Drying time | RAW | (For reference) | - | 533.243 | < 0.001 |
|  | 4H | 8.828 | 0.422 | - | < 0.001 |
|  | 4.5H | 15.824 | 0.422 | - | < 0.001 |
|  | 5H | 12.399 | 0.422 | - | < 0.001 |
|  | 5.5H | 20.324 | 0.422 | - | < 0.001 |
|  | 6H | 11.126 | 0.422 | - | < 0.001 |
| **Random effects** | | | | | |
| **Parameters** | **Variance component** | | | | |
| Day | 0.000 | | | | |
| Day×batches | 0.000 | | | | |
| Residual | 2.665 | | | | |

Table S7. Linear mixed model analysis results for drying time on pork jerky absorbance (dimensionless).

| **Parameters** | **Levels** | **Estimation** | **Standard Error** | ***F* value** | **significance** |
| --- | --- | --- | --- | --- | --- |
| **Fixed effects** | | | | | |
| Intercept | - | 16.542 | 0.359 | - | < 0.001 |
| Drying time | RAW | (For reference) | - | 2597.903 | < 0.001 |
|  | 4H | 31.719 | 0.508 | - | < 0.001 |
|  | 4.5H | 29.713 | 0.508 | - | < 0.001 |
|  | 5H | 31.251 | 0.508 | - | < 0.001 |
|  | 5.5H | 53.012 | 0.508 | - | < 0.001 |
|  | 6H | 46.329 | 0.508 | - | < 0.001 |
| **Random effects** | | | | | |
| **Parameters** | **Variance component** | | | | |
| Day | 0.000 | | | | |
| Day×batches | 0.000 | | | | |
| Residual | 3.872 | | | | |

Table S8. Influence of drying time on the content of free amino acids in pork jerky. (mg/100 g dry weight)

| **Drying time** | **RAW** | **4H** | **4.5H** | **5H** | **5.5H** | **6H** | **significance** |
| --- | --- | --- | --- | --- | --- | --- | --- |
| Aspartic | 41.317±1.345a | 4.68±0.998d | 5.174±1.536cd | 4.807±1.463d | 9.314±1.158b | 5.811±2.71c | ＜0.001 |
| Threonine | 41.203±1.092d | 70.608±3.35a | 68.348±1.371b | 65.07±2.251c | 70.754±1.174a | 68.461±2.592b | ＜0.001 |
| Serine | 26.394±0.783e | 36.751±1.534c | 37.077±2.833c | 35.404±2.601d | 44.47±2.68a | 39.349±3.621b | ＜0.001 |
| Glycine | 32.022±1.075e | 40.783±1.715d | 43.982±1.219c | 45.829±2.063b | 53.8±2.678a | 46.554±3.419b | ＜0.001 |
| Alanine | 88.055±2.554e | 161.329±6.238b | 166.698±5.137a | 154.349±3.114d | 155.988±0.407cd | 157.067±1.836c | ＜0.001 |
| Cystine | 17.571±1.789e | 19.449±0.676d | 21.129±1.822c | 22.823±0.879b | 26.242±1.109a | 22.765±2.882b | 0.014 |
| Valine | 35.108±1.207d | 53.871±1.217b | 52.008±0.571c | 54.398±1.686b | 57.983±1.54a | 54.408±3.037b | ＜0.001 |
| Methionine | 17.893±0.558d | 26.198±0.628bc | 27.241±1.083b | 26.029±1.348c | 30.68±0.957a | 26.866±3.99c | ＜0.001 |
| Isoleucine | 24.601±1.43e | 35.672±0.428d | 38.551±0.998c | 38.559±0.863c | 44.406±1.508a | 41.115±2.412b | ＜0.001 |
| Leucine | 43.237±2.189e | 60.075±1.662d | 64.075±1.477c | 63.441±2.231c | 75.062±2.971a | 72.535±2.051b | ＜0.001 |
| Tyrosine | 19.27±1.233a | 12.468±0.72d | 13.609±1.597c | 12.287±2.012e | 17.628±1.258b | 13.945±3.121c | 0.002 |
| Phenylalanine | 45.835±1.294a | 29.083±0.971d | 27.475±1.842e | 26.875±1.935e | 33.872±1.735b | 30.278±2.77c | ＜0.001 |
| Lysine | 6.017±1.29e | 68.582±2.041d | 70.175±1.831c | 73.02±4.679b | 80.158±1.128a | 80.912±3.554a | ＜0.001 |
| Arginine | 443.458±13.408a | 19.694±0.876c | 20.617±1.606c | 18.408±1.094c | 23.687±1.736b | 19.471±2.275c | ＜0.001 |
| Glutamate | 836.82±12.011f | 951.385±17.719e | 1052.881±36.91d | 1144.285±15.87c | 1200.552±16.25b | 1286.204±34.15a | ＜0.001 |
| Proline | 22.342±1.392e | 28.264±0.378bc | 27.39±1.308c | 24.764±2.02d | 32.657±1.771a | 28.519±3.069b | 0.017 |
| Histidine | 248.201±4.172f | 451.86±4.173a | 433.708±6.304d | 407.716±5.474e | 415.155±11.842c | 446.798±12.54b | ＜0.001 |

Table S9. The information of volatile organic compounds obtained from GC-IMS.

| **No.** | **Compound** | **CAS** | **RI^a^** | **Rt^b^ [sec]** | **Dt^c^ [RIPerl]** | **Peak volume** | | | | | | **significance** |
| --- | --- | --- | --- | --- | --- | --- | --- | --- | --- | --- | --- | --- |
|  |  |  |  |  |  | **RAW** | **4H** | **4.5H** | **5H** | **5.5H** | **6H** |  |
| **Alcohols** | | | | | | | | | | | | |
| 1 | 1-Octanol | 111-87-5 | 1058.3 | 904.507 | 1.46192 | 24.714±1.71f | 259.507±4.183a | 163.296±11.463e | 205.713±5.201c | 229.352±10.848b | 193.797±2.049d | ＜0.001 |
| 2 | 2-Heptanol | 543-49-7 | 905.8 | 535.405 | 1.40472 | 54.695±2.067e | 597.349±18.666a | 556.477±7.234c | 568.51±13.436b | 558.581±13.005c | 516.25±12.549d | ＜0.001 |
| 3 | Furfuryl alcohol | 98-00-0 | 894.5 | 514.494 | 1.35839 | 55.543±1.308f | 807.797±23.984a | 787.365±10.683b | 716.714±13.226e | 757.784±41.026c | 745.694±23.917d | ＜0.001 |
| 4 | n-Hexanol M | 111-27-3 | 877.5 | 482.673 | 1.32932 | 116.422±0.711e | 451.469±25.226a | 303.433±6.051d | 306.398±9.651d | 354.188±1.548b | 323.28±4.16c | ＜0.001 |
| 5 | n-Hexanol D | 111-27-3 | 877 | 481.763 | 1.64546 | 10.462±1.274f | 116.242±1.073a | 43.953±0.401c | 29.579±2.051e | 50.672±2.324b | 30.812±3.401d | ＜0.001 |
| 6 | 3-Methylpentanol | 589-35-5 | 852.5 | 439.032 | 1.30025 | 24.892±1.847d | 504.656±12.925b | 454.223±27.535c | 525.803±29.024a | 531.772±13.366a | 506.371±22.782b | ＜0.001 |
| 7 | (E)-2-Hexen-1-ol | 2305-21-7 | 833.9 | 409.029 | 1.18761 | 28.592±2.702f | 48.759±0.663c | 44.589±0.59d | 92.289±2.235a | 84.979±0.74b | 41.011±2.386e | ＜0.001 |
| 8 | 1-Pentanol M | 71-41-0 | 770.7 | 319.683 | 1.25724 | 556.953±32.105a | 431.128±12.329d | 445.724±10.118c | 399.952±15.866e | 406.487±14.926e | 468.478±11.065b | ＜0.001 |
| 9 | 1-Pentanol D | 71-41-0 | 770.4 | 319.234 | 1.51558 | 381.452±3.273a | 350.91±5.048c | 316.803±14.371e | 328.378±23.571d | 358.699±4.846b | 362.442±10.709b | ＜0.001 |
| 10 | 3-Methylbutanol | 123-51-3 | 755.6 | 299.502 | 1.10738 | 30.941±2.085e | 98.297±0.922c | 81.897±0.281d | 120.157±5.224a | 120.579±5.062a | 116.733±0.977b | ＜0.001 |
| 11 | 3-Methyl-3-buten-1-ol | 763-32-6 | 749.6 | 291.879 | 1.40711 | 24.055±1.691f | 202.782±1.061b | 139.086±2.437e | 210.439±8.351a | 188.358±6.723c | 169.088±5.054d | ＜0.001 |
| 12 | 2-Methylbutanol | 137-32-6 | 718.7 | 255.554 | 1.23012 | 40.73±2.259e | 229.573±2.946b | 216.324±11.612c | 204.234±10.775d | 214.124±14.671c | 242.976±0.624a | ＜0.001 |
| **Aldehydes** | | | | | | | | | | | | |
| 13 | Nonanal M | 124-19-6 | 1103.2 | 1049.964 | 1.47924 | 392.563±13.066d | 1619.63±21.467a | 1382.15±14.726b | 1249.89±72.288c | 1620.604±47.769a | 1357.815±81.745b | ＜0.001 |
| 14 | Nonanal D | 124-19-6 | 1104.2 | 1053.556 | 1.94688 | 50.256±2.745e | 364.571±16.673a | 230.664±4.034c | 231.572±3.897c | 345.856±8.21b | 190.068±9.16d | ＜0.001 |
| 15 | (E)-2-Octenal M | 2548-87-0 | 1059.5 | 908.099 | 1.33753 | 107.184±1.46f | 630.104±3.376a | 351.728±0.76e | 443.462±12.155d | 550.057±13.627b | 514.621±8.084c | ＜0.001 |
| 16 | (E)-2-Octenal D | 2548-87-0 | 1059.5 | 908.099 | 1.82249 | 13.249±1.915e | 50.007±3.223a | 16.199±0.966d | 31.299±2.94c | 43.286±1.056b | 31.675±1.291c | ＜0.001 |
| 17 | Octanal | 124-13-0 | 1010.2 | 770.883 | 1.41108 | 139.336±1.215f | 307.152±7.097d | 398.869±6.026c | 256.688±12.043e | 415.028±5.151b | 445.548±9.325a | ＜0.001 |
| 18 | (E)-2-Heptenal M | 18829-55-5 | 968.8 | 668.146 | 1.25483 | 164.108±6.709e | 540.938±11.343b | 382.32±8.648d | 538.412±28.936b | 576.04±3.048a | 519.878±7.426c | ＜0.001 |
| 19 | 5-Methyl furfural | 620-02-0 | 970 | 670.873 | 1.38201 | 28.64±0.627d | 520.785±10.841a | 478.516±29.361b | 523.614±14.249a | 527.248±15.718a | 440.337±10.883c | ＜0.001 |
| 20 | (E)-2-Heptenal D | 18829-55-5 | 967.6 | 665.418 | 1.66999 | 41.916±0.728f | 580.432±11.072b | 237.881±1.542e | 556.607±12.34c | 623.754±13.526a | 494.171±16.249d | ＜0.001 |
| 21 | Heptanal M | 111-71-7 | 906.8 | 537.224 | 1.33841 | 234.757±2.054f | 594.631±4.094c | 569.683±20.513e | 588.452±5.698d | 627.476±7.366a | 603.732±16.066b | ＜0.001 |
| 22 | Heptanal D | 111-71-7 | 906.3 | 536.314 | 1.69452 | 59.488±1.514f | 865.38±47.583c | 684.335±18.39d | 655.837±22.44e | 911.813±9.225a | 889.475±21.083b | ＜0.001 |
| 23 | 2-Hexenal M | 505-57-7 | 853.6 | 440.85 | 1.18216 | 50.849±0.703e | 183.17±6.996c | 131.09±2.184d | 191.607±4.34b | 234.005±7.274a | 182.172±7.699c | ＜0.001 |
| 24 | 2-Hexenal D | 505-57-7 | 852 | 438.123 | 1.51555 | 15.491±0.451f | 164.534±2.183d | 92.523±4.078e | 188.991±7.498b | 281.31±5.533a | 175.283±7.766c | ＜0.001 |
| 25 | Hexanal | 66-25-1 | 798.9 | 358.249 | 1.55555 | 2353.49±90.483b | 2296.85±54.775c | 2041.25±31.046d | 2268.35±35.035c | 2341.873±81.935b | 2415.241±85.224a | ＜0.001 |
| 26 | 2-Methylpentanal | 123-15-9 | 754.9 | 298.605 | 1.21728 | 30.011±1.939e | 296.381±13.168c | 236.085±6.457d | 300.468±2.07b | 308.518±3.74a | 298.299±2.907bc | ＜0.001 |
| 27 | (E)-2-Pentenal | 1576-87-0 | 754.9 | 298.605 | 1.35573 | 9.078±0.687f | 78.269±1.851d | 37.919±1.538e | 129.447±1.668b | 146.304±3.247a | 110.657±4.534c | ＜0.001 |
| 28 | Pentanal M | 110-62-3 | 666.9 | 209.364 | 1.1873 | 295.793±4.667a | 100.13±2.074c | 110.689±1.486b | 79.401±2.741e | 88.705±2.564d | 88.716±3.056d | ＜0.001 |
| 29 | Pentanal D | 110-62-3 | 664.9 | 208.018 | 1.40425 | 463.837±7.967e | 836.335±17.101c | 825.077±16.69d | 836.828±4.036c | 873.681±18.708b | 938.163±17.618a | ＜0.001 |
| 30 | Butanal M | 123-72-8 | 581.5 | 159.137 | 1.11879 | 107.122±1.631f | 156.041±9.209a | 148.632±1.81b | 128.647±5.516d | 125.386±2.233e | 146.047±3.9c | ＜0.001 |
| 31 | Butanal D | 123-72-8 | 579.7 | 158.23 | 1.28337 | 35.994±2.137f | 266.333±5.09e | 313.293±2.185d | 392.213±11.65c | 452.663±6.357b | 541.116±16.009a | ＜0.001 |
| **Ketones** | | | | | | | | | | | | |
| 32 | Furaneol | 3658-77-3 | 1087.9 | 997.887 | 1.22259 | 759.191±27.537a | 265.482±6.871d | 277.239±9.134c | 276.724±8.98c | 303.942±6.096b | 309.965±12.719b | ＜0.001 |
| 33 | 2-Heptanone M | 110-43-0 | 895 | 515.403 | 1.26028 | 101.617±1.483f | 414.547±10.229a | 396.269±7.505b | 322.84±7.94e | 369.36±9.011d | 386.378±10.366c | ＜0.001 |
| 34 | 2-Heptanone D | 110-43-0 | 894.5 | 514.494 | 1.63274 | 16.666±2.125e | 529.674±31.957a | 421.929±6.394b | 253.582±12.848d | 370.476±14.33c | 419.265±7.917b | ＜0.001 |
| 35 | 2-Hexanone | 591-78-6 | 785.7 | 340.76 | 1.49988 | 17.657±1.332e | 67.956±3.83b | 76.89±0.935a | 37.62±2.242d | 59.444±1.377c | 59.213±5.638c | ＜0.001 |
| 36 | 2,3-Pentanedione | 600-14-6 | 705.8 | 241.652 | 1.29292 | 257.112±8.768a | 202.269±0.179d | 189.963±4.04e | 192.612±7.93e | 208.761±2.713c | 232.075±2.881b | ＜0.001 |
| 37 | 2-Butanone | 78-93-3 | 612.4 | 175.73 | 1.24582 | 461.013±4.818e | 424.123±1.27f | 486.244±6.893d | 542.126±21.202c | 629.555±19.375a | 564.012±5.361b | ＜0.001 |
| 38 | 2,3-Butanedione | 431-03-8 | 583.6 | 160.248 | 1.19355 | 59.633±3.354f | 152.458±4.381e | 182.884±8.938d | 190.535±0.784d | 212.482±3.122a | 202.478±5.36b | ＜0.001 |
| **Acids** | | | | | | | | | | | | |
| 39 | Hexanoic acid | 142-62-1 | 1027.1 | 815.433 | 1.29721 | 22.758±2.237f | 165.538±2.693a | 66.649±1.864d | 114.715±0.749b | 82.983±3.028c | 48.283±2.021e | ＜0.001 |
| 40 | Pentanoic acid | 109-52-4 | 913.4 | 549.952 | 1.21577 | 18.327±1.449f | 240.757±8.312e | 275.446±4.344d | 329.341±15.992a | 318.248±3.825b | 301.467±14.445c | ＜0.001 |
| **Esters** | | | | | | | | | | | | |
| 41 | Furanmethanol acetate | 623-17-6 | 994.7 | 731.788 | 1.4347 | 152.302±6.957e | 618.93±32.702a | 483.319±10.172d | 530.51±11.991c | 592.377±6.256b | 591.801±19.216b | ＜0.001 |
| 42 | Butyl acetate | 123-86-4 | 815.5 | 381.569 | 1.26438 | 33.791±1.592f | 270.177±8.484c | 168.888±5.689e | 195.216±7.953d | 273.793±7.135b | 300.182±4.766a | ＜0.001 |
| 43 | Isopentyl formate | 110-45-2 | 785.7 | 340.76 | 1.29435 | 33.175±0.892f | 302.306±4.947b | 312.814±0.237a | 253.735±7.151e | 278.073±3.865c | 259.753±12.859d | ＜0.001 |
| 44 | Ethyl propanoate | 105-37-3 | 704.5 | 240.307 | 1.41995 | 367.447±6.472e | 798.576±4.39b | 753.748±10.146c | 719.595±14.898d | 754.729±2.282c | 926.658±30.889a | ＜0.001 |
| **Heterocyclic compounds** | | | | | | | | | | | | |
| 45 | 2-Methoxy-3-isobutyl pyrazine | 24683-00-9 | 1183.6 | 1371.407 | 1.82407 | 370.515±3.504b | 382.781±0.917a | 357.106±1.415d | 357.13±2.083d | 363.444±1.641c | 339.174±2.187e | ＜0.001 |
| 46 | Acetyl pyrazine | 22047-25-2 | 1026.9 | 814.719 | 1.2163 | 31.988±0.881f | 206.161±9.701a | 81.982±5.782d | 153.109±4.848b | 102.58±3.013c | 61.871±2.745e | ＜0.001 |
| 47 | 2-Ethyl-6-methylpyrazine | 13925-03-6 | 1009.9 | 769.974 | 1.21587 | 60.791±1.533f | 798.55±7.932a | 321.811±3.547d | 536.566±9.489b | 409.001±14.096c | 225.991±5.037e | ＜0.001 |
| 48 | Trimethyl-pyrazine | 14667-55-1 | 993.6 | 729.061 | 1.15945 | 232.001±2.32f | 630.763±6.17a | 417.044±3.404e | 476.816±12.811d | 541.936±20.11b | 505.538±11.807c | ＜0.001 |
| 49 | 2-Pentylfuran | 3777-69-3 | 997.9 | 739.971 | 1.25301 | 21.982±1.377f | 351.023±5.096a | 253.35±6.534d | 249.218±5.966e | 326.715±10.895b | 317.691±7.765c | ＜0.001 |
| 50 | Ethyl pyrazine | 13925-00-3 | 930.8 | 584.501 | 1.16399 | 31.383±1.755e | 308.848±8.106a | 301.782±22.672b | 307.769±4.374a | 292.268±9.488c | 139.994±4.503d | ＜0.001 |
| 51 | 2,5-Dimethyl pyrazine | 123-32-0 | 913.9 | 550.861 | 1.08677 | 27.939±0.921f | 126.305±4.443e | 137.692±2.856d | 166.063±6.883c | 169.469±1.056b | 188.996±5.141a | ＜0.001 |
| 52 | 2-Ethylfuran | 3208-16-0 | 721.6 | 258.693 | 1.33146 | 83.437±3.489e | 404.851±17.898c | 477.258±14.677b | 393.367±18.943d | 395.557±8.954d | 510.569±11.996a | ＜0.001 |
| **Others** | | | | | | | | | | | | |
| 53 | 1,2-Dimethoxyethane | 110-71-4 | 668.3 | 210.261 | 1.28436 | 304.817±13.012a | 273.015±4.222c | 286.73±0.065b | 275.68±8.541c | 276.684±2.474c | 273.736±3.553c | ＜0.001 |

M, monomers; D, dimers.

a. Represents the retention index in the capillary GC column.

b. Represents the retention time in the capillary GC column.

c. Represents the drift time in the drift tube.

Table S10. Top 10 VOCs identified by SVR-SHAP for the prediction of pork jerky physicochemical indices.

| **Rank** | **VOCs** | **Mean \|SHAP value\|** | **Contribution (%)** |
| --- | --- | --- | --- |
| **Moisture** | | | |
| 1 | 1,2-Dimethoxyethane | 0.0786 | 19.44 |
| 2 | 1-Pentanol M | 0.0565 | 13.97 |
| 3 | Pentanal M | 0.0392 | 9.70 |
| 4 | (E)-2-Octenal D | 0.0366 | 9.06 |
| 5 | Furaneol | 0.0357 | 8.83 |
| 6 | 1-Pentanol D | 0.0357 | 8.82 |
| 7 | 3-Methylpentanol | 0.0350 | 8.67 |
| 8 | Hexanal | 0.0334 | 8.27 |
| 9 | Ethyl propanoate | 0.0271 | 6.70 |
| 10 | 2-Ethylfuran | 0.0265 | 6.55 |
| **Absorbance** | | | |
| 1 | Octanal | 0.0550 | 13.03 |
| 2 | 2-Hexanone | 0.0538 | 12.73 |
| 3 | 2-Butanone | 0.0459 | 10.87 |
| 4 | 2-Hexenal M | 0.0414 | 9.80 |
| 5 | 2-Hexenal D | 0.0406 | 9.63 |
| 6 | 1-Pentanol D | 0.0405 | 9.59 |
| 7 | Butyl acetate | 0.0397 | 9.40 |
| 8 | Acetylpyrazine | 0.0360 | 8.53 |
| 9 | (E)-2-Pentenal | 0.0348 | 8.24 |
| 10 | Heptanal D | 0.0345 | 8.18 |
| **Fluorescence intensity** | | | |
| 1 | Hexanal | 0.0844 | 17.28 |
| 2 | 2-Hexanone | 0.0613 | 12.54 |
| 3 | 2-Butanone | 0.0557 | 11.41 |
| 4 | 2-Hexen-1-ol | 0.0451 | 9.24 |
| 5 | Octanal | 0.0449 | 9.20 |
| 6 | 2-Hexenal M | 0.0425 | 8.69 |
| 7 | Ethyl propanoate | 0.0403 | 8.24 |
| 8 | 2-Hexenal D | 0.0397 | 8.14 |
| 9 | 1-Pentanol D | 0.0385 | 7.89 |
| 10 | Ethylpyrazine | 0.0359 | 7.35 |
| ***L** value** | | | |
| 1 | Hexanal | 0.1086 | 17.66 |
| 2 | 2-Butanone | 0.0683 | 11.11 |
| 3 | 2-Hexenal M | 0.0663 | 10.78 |
| 4 | 3-Isobutyl-2-methoxypyrazine | 0.0622 | 10.12 |
| 5 | 1-Pentanol D | 0.0559 | 9.10 |
| 6 | (E)-2-Pentenal | 0.0559 | 9.09 |
| 7 | 2-Hexen-1-ol | 0.0545 | 8.87 |
| 8 | (E)-2-Octenal D | 0.0520 | 8.46 |
| 9 | 2-Hexanone | 0.0456 | 7.41 |
| 10 | 2-Hexenal D | 0.0455 | 7.40 |
| ***a** value** | | | |
| 1 | Hexanal | 0.1764 | 16.67 |
| 2 | 2-Hexanone | 0.1559 | 14.73 |
| 3 | 1,2-Dimethoxyethane | 0.1173 | 11.08 |
| 4 | Acetylpyrazine | 0.1076 | 10.17 |
| 5 | 2-Hexen-1-ol | 0.0934 | 8.82 |
| 6 | Hexanoic acid | 0.0902 | 8.52 |
| 7 | Ethyl propanoate | 0.0824 | 7.79 |
| 8 | n-Hexanol D | 0.0807 | 7.62 |
| 9 | 2-Hexenal M | 0.0794 | 7.50 |
| 10 | Hexamethyldisiloxane | 0.0753 | 7.11 |
| ***b** value** | | | |
| 1 | 1-Pentanol D | 0.0866 | 16.57 |
| 2 | 2-Butanone | 0.0636 | 12.18 |
| 3 | Hexanal | 0.0562 | 10.75 |
| 4 | 2-Hexanone | 0.0554 | 10.61 |
| 5 | 3-Isobutyl-2-methoxypyrazine | 0.0523 | 10.01 |
| 6 | 2-Hexenal M | 0.0434 | 8.31 |
| 7 | (E)-2-Octenal D | 0.0422 | 8.08 |
| 8 | 2,3-Pentanedione | 0.0417 | 7.99 |
| 9 | 1,2-Dimethoxyethane | 0.0411 | 7.88 |
| 10 | n-Hexanol D | 0.0399 | 7.63 |

Note: Mean |SHAP value| represents the average absolute contribution of each VOC to the SVR prediction model. Contribution (%) was calculated based on the sum of the mean |SHAP value| values of the top 10 VOCs for each physicochemical index. VOCs were ranked according to mean |SHAP value|. M and D represent monomers and dimers, respectively.

Table S11. Top 10 VOCs identified by ETR-SHAP for the prediction of pork jerky physicochemical indices.

| **Rank** | **VOCs** | **Mean \|SHAP value\|** | **Contribution (%)** |
| --- | --- | --- | --- |
| **Moisture** | | | |
| 1 | Heptanal M | 0.0341 | 12.40 |
| 2 | Furfuryl alcohol | 0.0322 | 11.70 |
| 3 | Isopentyl formate | 0.0297 | 10.79 |
| 4 | 2-Heptanol | 0.0274 | 9.95 |
| 5 | Furaneol | 0.0270 | 9.83 |
| 6 | 5-Methylfurfural | 0.0266 | 9.66 |
| 7 | n-Hexanol M | 0.0251 | 9.12 |
| 8 | Nonanal M | 0.0246 | 8.95 |
| 9 | Pentanal M | 0.0245 | 8.92 |
| 10 | Ethyl propanoate | 0.0239 | 8.68 |
| **Absorbance** | | | |
| 1 | Heptanal D | 0.0409 | 14.17 |
| 2 | Heptanal M | 0.0327 | 11.32 |
| 3 | Furaneol | 0.0301 | 10.43 |
| 4 | Butanal D | 0.0290 | 10.03 |
| 5 | 2,3-Butanedione | 0.0272 | 9.41 |
| 6 | 2-Pentylfuran | 0.0270 | 9.35 |
| 7 | Butyl acetate | 0.0266 | 9.20 |
| 8 | 2,5-Dimethylpyrazine | 0.0256 | 8.86 |
| 9 | Pentanal D | 0.0250 | 8.64 |
| 10 | 5-Methylfurfural | 0.0248 | 8.59 |
| **Fluorescence intensity** | | | |
| 1 | 2-Hexenal D | 0.0488 | 15.31 |
| 2 | 2-Hexenal M | 0.0366 | 11.50 |
| 3 | n-Hexanol D | 0.0359 | 11.26 |
| 4 | Isopentyl formate | 0.0320 | 10.05 |
| 5 | Nonanal D | 0.0298 | 9.34 |
| 6 | Heptanal M | 0.0284 | 8.92 |
| 7 | Hexanoic acid | 0.0279 | 8.75 |
| 8 | Ethyl propanoate | 0.0278 | 8.74 |
| 9 | 5-Methylfurfural | 0.0264 | 8.29 |
| 10 | 2-Ethyl-6-methylpyrazine | 0.0250 | 7.84 |
| ***L** value** | | | |
| 1 | Hexamethyldisiloxane | 0.0875 | 13.15 |
| 2 | (E)-2-Pentenal | 0.0850 | 12.76 |
| 3 | 2-Butanone | 0.0793 | 11.91 |
| 4 | 2,3-Butanedione | 0.0749 | 11.25 |
| 5 | Pentanoic acid | 0.0663 | 9.96 |
| 6 | 2-Hexen-1-ol | 0.0638 | 9.59 |
| 7 | Butanal D | 0.0567 | 8.52 |
| 8 | 2,5-Dimethylpyrazine | 0.0557 | 8.37 |
| 9 | 3-Methylbutanol | 0.0505 | 7.59 |
| 10 | 3-Isobutyl-2-methoxypyrazine | 0.0460 | 6.91 |
| ***a** value** | | | |
| 1 | 3-Isobutyl-2-methoxypyrazine | 0.0914 | 19.48 |
| 2 | Butanal D | 0.0705 | 15.01 |
| 3 | Ethyl propanoate | 0.0695 | 14.81 |
| 4 | Hexamethyldisiloxane | 0.0438 | 9.33 |
| 5 | Ethylpyrazine | 0.0363 | 7.74 |
| 6 | 2-Ethylfuran | 0.0363 | 7.74 |
| 7 | (E)-2-Pentenal | 0.0352 | 7.50 |
| 8 | Pentanal D | 0.0296 | 6.31 |
| 9 | 1,2-Dimethoxyethane | 0.0284 | 6.04 |
| 10 | 3-Methylbutanol | 0.0283 | 6.03 |
| ***b** value** | | | |
| 1 | 2-Butanone | 0.1103 | 20.65 |
| 2 | 2,3-Butanedione | 0.0656 | 12.27 |
| 3 | Octanal | 0.0592 | 11.08 |
| 4 | 3-Isobutyl-2-methoxypyrazine | 0.0495 | 9.27 |
| 5 | Pentanoic acid | 0.0474 | 8.87 |
| 6 | Heptanal M | 0.0458 | 8.57 |
| 7 | 2,5-Dimethylpyrazine | 0.0404 | 7.56 |
| 8 | 2-Hexenal M | 0.0397 | 7.43 |
| 9 | Hexanal | 0.0391 | 7.32 |
| 10 | Isopentyl formate | 0.0373 | 6.99 |
